# *Gβγ* activates *PIP2* hydrolysis by recruiting and orienting *PLCβ* on the membrane surface

**DOI:** 10.1073/pnas.2301121120

**Published:** 2023-05-12

**Authors:** Maria E. Falzone, Roderick MacKinnon

**Affiliations:** ^a^Laboratory of Molecular Neurobiology and Biophysics, The Rockefeller University, New York, NY 10065; ^b^HHMI, The Rockefeller University, New York, NY 10065

**Keywords:** *PLCβ*, *Gβγ*, *PIP2*, GPCR signaling, membrane recruitment

## Abstract

*Phospholipase C-βs (PLCβs)* catalyze the hydrolysis of phosphatidylinositol 4, 5–bisphosphate (PIP2) into inositoltriphosphate (IP3) and diacylglycerol  (DAG). PIP2 regulates the activity of many membrane proteins, while *IP3* and *DAG* lead to increased intracellular Ca^2+^ levels and activate protein kinase C, respectively. *PLCβs* are regulated by G protein–coupled receptors through direct interaction with ${G\alpha }_{q}$ and Gβγ and are aqueous-soluble enzymes that must bind to the cell membrane to act on their lipid substrate. This study addresses the mechanism by which Gβγ activates *PLCβ*3. We show that *PLCβ*3 functions as a slow Michaelis–Menten enzyme ( ${k_{\mathrm{cat}}∼2\;s^{-1},\;K}_{M}∼0.43\;mol\%$ ) on membrane surfaces. We used membrane partitioning experiments to study the solution-membrane localization equilibrium of *PLCβ*3. Its partition coefficient is such that only a small quantity of *PLCβ*3 exists in the membrane in the absence of Gβγ . When Gβγ is present, equilibrium binding on the membrane surface increases *PLCβ*3 in the membrane, increasing $V_{\max}$ in proportion. Atomic structures on membrane vesicle surfaces show that two Gβγ anchor *PLCβ*3 with its catalytic site oriented toward the membrane surface. Taken together, the enzyme kinetic, membrane partitioning, and structural data show that Gβγ activates *PLCβ* by increasing its concentration on the membrane surface and orienting its catalytic core to engage PIP2 . This principle of activation explains rapid stimulated catalysis with low background activity, which is essential to the biological processes mediated by PIP2*, IP3*, and *DAG*.

## Significance

GPCRs are major mediators of transmembrane signal transduction, responding to a wide range of stimuli including hormones and neurotransmitters. Important targets of GPCR signaling, *PLCβ* enzymes catalyze the hydrolysis of PIP2 into *IP3* and *DAG*, leading to increased intracellular Ca^2+^ levels and activation of PKC, respectively. *PLCβ*s exhibit very low basal activity through multiple mechanisms of autoinhibition and are activated by both ${G\alpha }_{q}$ and Gβγ . In this study, we demonstrate that Gβγ activates *PLCβ* by recruiting it to the membrane where its substrate PIP2 resides and by orienting its active site. This activation mechanism permits robust and rapid activation of *PLCβ* upon GPCR stimulation in the setting of low background activity during GPCR quiescence.

**Fig. 1.**
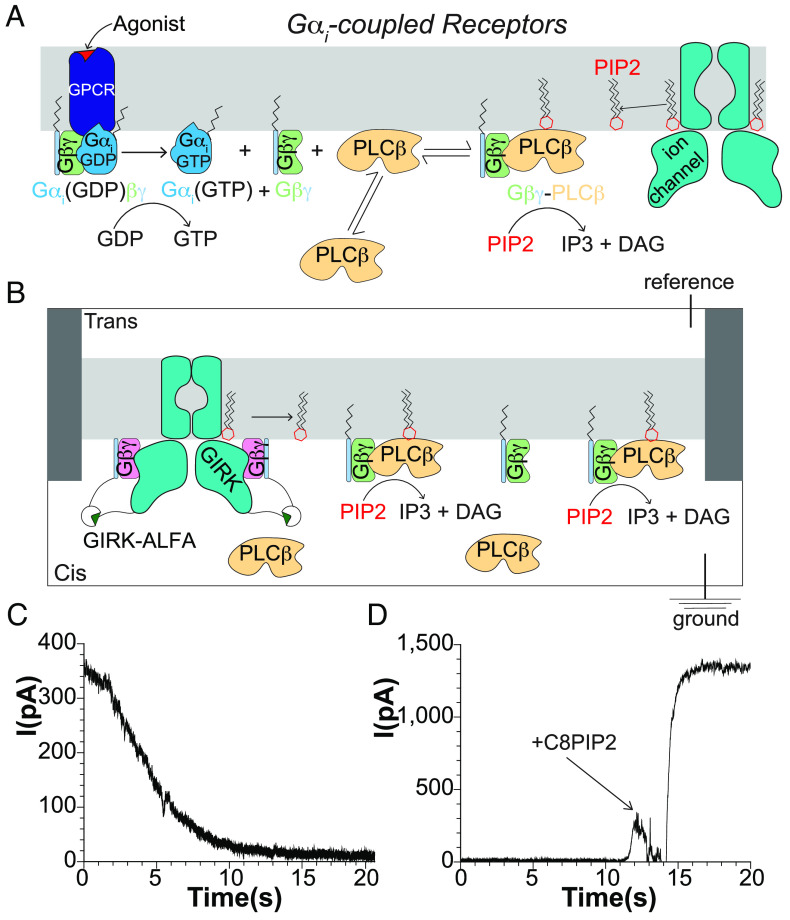
Development of a planar lipid bilayer assay for *PLCβ* activity using a PIP2 -dependent ion channel as readout. (*A*) Cartoon summary of ${G\alpha }_{i}$-dependent signaling to *PLCβ* through Gβγ . (*B*) Cartoon schematic of planar lipid bilayer setup used to measure *PLCβ* function. (*C*) Representative current decay upon *PLCβ*-dependent depletion of PIP2 . (*D*) Representative current recovery upon reactivation of incorporated channels with short-chain C8PIP2. This experiment was carried out under subsaturating long-chain PIP2 (1.0 mol% ) in the bilayer, which correlates to ~30% of maximal GIRK current. In *D*, saturating C8PIP2 was added, which leads to ~3× the amount of starting current.

Phospholipase C-β (*PLCβ*) enzymes cleave phosphatidylinositol 4,5-bisphosphate ( PIP2 ) into inositoltriphosphate ( IP3 ) and diacylglycerol ( DAG ) (1, 2). Their activity is controlled by G protein–coupled receptors (GPCRs) through direct interaction with G proteins (3–5). IP3 increases intracellular calcium, DAG activates protein kinase C, and levels of PIP2 regulate numerous ion channels. Therefore, the **PLCβ* *enzymes under GPCR regulation are central to cellular signaling (Fig. 1*A*) (6–8). There are four *PLCβs* (1–4) in humans: PLCβ4 is activated by ${G\alpha }_{q,}$ and *PLCβ*1–3 are activated by both ${G\alpha }_{q}$ and Gβγ. *PLCβ*2/3 are also activated by the small GTPases Rac1/2 (9–15).

What do we know about *PLCβs* and their regulation by G proteins? **PLCβ*s* are cytoplasmic enzymes that must access the membrane where their substrate PIP2 resides in the inner leaflet. They contain a catalytic core, a proximal C-terminal domain (CTD) with autoinhibitory activity, and a distal CTD with structural homology to a bin-amphiphysin-Rvs domain important for membrane binding (3, 4). At the active site, an X–Y linker exerts additional autoinhibitory regulation by direct occlusion (9, 15–17). ${G\alpha }_{q}$ binds to the proximal and distal CTDs, displacing the autoinhibitory proximal CTD from the catalytic core and Rac1 binds to the PH domain of *PLCβ*2 (9, 18–21). Notably, in both cases the autoinhibitory X–Y linker still occludes the active site. Less is known about regulation of *PLCβs* by Gβγ . Potential binding sites have been described, but no structures have been determined (3, 4). The focus of this study is regulation of *PLCβ*3 by Gβγ.

The mechanism of *PLCβ* activation by Gβγ is unknown. In vitro studies have concluded that locally concentrating *PLCβ* on the membrane is not the basis of activation and this still dominates thinking in the field (3, 4, 22–28). However, the requirement of the lipid group on Gβγ to achieve activation and the demonstration that over expression of G proteins in cells increases *PLCβ* in the membrane fraction suggests that a localization mechanism needs revisiting (13, 29). Part of the challenge in characterizing *PLCβ* enzymes is precisely the membrane involvement. *PLCβs* reside in 3 dimensions (the cytoplasm) but catalyze on a two-dimensional surface (the membrane). Functional measurements must account for this and at the same time permit sufficient time resolution, unlike the standard radioactive assay used in the field until now. To overcome the challenge, we have developed new functional methods, including a rapid kinetic analysis of *PLCβ*3 enzyme activity that employs a direct read-out of PIP2 concentration as a function of time, a membrane partitioning assay to quantify membrane recruitment, and atomic structures on lipid membrane surfaces, to analyze the mechanism by which Gβγ activates *PLCβs*.

## Results

To explain with accuracy our data analysis, we present a series of equations and their rationale. At least a qualitative understanding of these equations is required to fully appreciate the meaning and wider significance of the data, and what it implies about the molecular mechanisms crucial for *PLCβ*3 function. Some of the analysis and associated equations are, to our knowledge, unfamiliar to biochemical analysis. In particular, when analyzing both the kinetics of PIP2 hydrolysis on a membrane surface and the equilibrium binding reaction between proteins on a membrane surface, we encountered the complex issue of processes occurring in 2 dimensions that involve components in 3 dimensions. We dealt with this issue in a particular way, which we describe thoroughly to stimulate debate and invite critique. We appreciate that many readers will want to grasp the biological implications of this work without getting bogged down by equations. For this reason, we have explained the meaning of each equation in words, which should be sufficient to understand the main conclusions of this work.

### Development of a Planar Lipid Bilayer Assay for *PLCβ*3 Function.

We developed a detergent-free, planar lipid bilayer assay to measure *PLCβ*3 function using a PIP2-dependent ion channel to report its concentration over time (Fig. 1 *B*–*D*). Briefly, two chamber cups were connected in the vertical configuration by a ~250 μm hole in a 100 μm piece of Fluorinated ethylene propylene copolymer (30). A ground electrode was placed in the Cis chamber and a reference electrode in the Trans chamber (Fig. 1*B*). Lipids dispersed in decane were used to paint a bilayer over the hole separating the two chambers. We used a 2:1:1 mixture of 1, 2-dioleoyl-sn-glycero-3-phosphoethanolamine (DOPE): 1-palmitoyl-2-oleoyl-glycero-3-phosphocholine (POPC): 1-palmitoyl-2-oleoyl-sn-glycero-3-phospho-L-serine (POPS) lipids and included a predetermined mole fraction of long-chain PIP2 inside the membrane to set its starting concentration. Ion channels and G proteins were incorporated by proteolipid vesicle application to the bilayer, and the current from reconstituted ion channels was measured (30). We added *PLCβ*3 to the Cis chamber, which was subjected to continuous mixing to ensure homogeneity of the chamber.

The PIP2-dependent, G protein-dependent inward rectifier K^+^ channel-2 (GIRK2, specified as GIRK) was used as a readout of PIP2 concentration. This channel is well characterized in vitro, strictly depends on PIP2 for channel opening, and is amenable to measuring large macroscopic currents using planar lipid bilayers (31, 32). Further, GIRK exhibits fast rates of association and disassociation of PIP2 , which permits the measurement of *PLCβ*3 catalytic activity that is not filtered by a slow channel response (31). Experiments were carried out in the presence of symmetric MgCl_2_ to ensure blockage of channels with their PIP2 binding sites facing the Trans chamber, which is not accessible to *PLCβ*3 (Fig. 1*B*) (33). This ensures that when positive voltage is applied to the reference relative to the ground, the current is derived only from channels accessible to *PLCβ*3 added to the Cis chamber.

GIRK also requires Gβγ for channel activity. To separate the effects of Gβγ on channel function and *PLCβ*3 activity we used the ALFA nanobody system (34) to tether soluble Gβγ to GIRK (35). We tagged GIRK with the short ALFA peptide on the C-terminus and Gγ with the ALFA nanobody on the N-terminus in the background of the C68S mutant, which prevents lipidation of Gγ . Nanobody-tagged Gγ assembled normally with Gβ and was able to bind to other effectors (35). Because the ALFA nanobody binds to the ALFA tag with ~30 pM affinity (34), at 30 nM concentration, the ALFA nanobody-tagged Gβγ fully activates ALFA peptide-tagged GIRK. In addition, the nanobody-tagged Gβγ does not activate *PLCβ*3 due to its lack of a lipid anchor (13, 29).

Human *PLCβ*3 was used to establish our assay owing to its significant activation by both Gβγ and ${G\alpha }_{q}$ (14, 36, 37). The addition of *PLCβ*3 to membranes already containing lipidated Gβγ , following an equilibration period of about 2 s, led to a rapid current decay that was complete in ~20 s (Fig. 1*C*). Subsequent addition of 32 μm C8PIP2, an aqueous-soluble, short chain version of PIP2, rescued the current to a maximum level (Fig. 1*D*)(31), indicating that the current decay was due to PIP2 depletion from the bilayer by the *PLCβ*3 enzyme. The *PLCβ*3 mediated current decay was slower than when C8PIP2 is rapidly removed by perfusion (31). Furthermore, the rate of *PLCβ*3-mediated current decay depends on the *PLCβ*3 concentration (*SI Appendix*, Fig. S1). These findings indicate that the decay measures the rate of *PLCβ*3 catalytic activity rather than PIP2 unbinding from the channel. No change in the current was observed following *PLCβ*3 addition in the absence of CaCl_2_ (2 mM EGTA), which is required for enzymatic function (*SI Appendix*, Fig. S1*A*). Repetitions of these experiments yielded consistent results with very similar time courses of current decay. These observations indicate that we can measure *PLCβ*3 catalytic activity using this system and that the addition of *PLCβ*3 does not induce artifacts to the bilayer or to reconstituted GIRK channels.

### Kinetic Analysis of PIP2 Hydrolysis by PLCβ3.

The interfacial nature of *PLCβ*3 activity presents a challenge to the study of its function because *PLCβ*3 is a soluble enzyme that must associate with the membrane to carry out catalysis. To describe the reaction occurring at the two-dimensional membrane surface, which must account for the exchange of *PLCβ*3 with the three-dimensional water phase, we give concentrations as dimensionless mole fraction × 100 ( mf , expressed as mol% ) using square brackets, [*quantity*], unless specified as molar units using square brackets with subscript *molar*, [*quantity*]*_molar_*. Furthermore, to simplify expressions, we approximate mf within each solvent phase, water or lipid, as moles solute per moles solvent rather than moles solute per moles solvent plus solute. This approximation introduces into the kinetic analysis a maximum error in mf of 1.0 % for the PIP2 concentration in membranes and less than 1.0 % for all other components. For PIP2 in membranes, the initial mf is predetermined through the bilayer lipid composition. For *PLCβ*3, the mf in membranes is calculated from that in three-dimensional solution using its partition coefficient, which is described below.

**Fig. 2.**
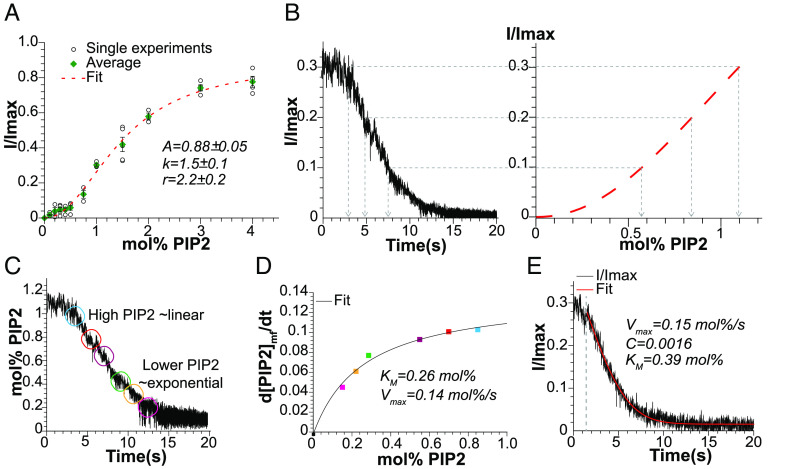
Extraction of values for kinetic parameters for *PLCβ*3 catalysis in the presence of lipidated Gβγ from current decay curves. (*A*) PIP2 activation curve for GIRK varying the mole% of PIP2 in the bilayer and maximally activating with C8PIP2. Green diamonds are average values, open circles are values from each experiment, and error bars are SEM. Each point is from 3–5 experiments. The normalized current (I/Imax) is fit to a modified hill equation, Eq. **1** (dashed red curve). R^2^ = 0.994. (*B*) Demonstration of using the PIP2 activation curve (*Right*) to convert the current decay (*Left*) to PIP2 decay. Points on the normalized current decay are matched to mol% PIP2 and time. (*C*) Resulting PIP2 decay over time. Circles denote regions used for measuring the rates graphed in *D*. (*D*) Plot of ${d[PIP2]}_{\mathrm{mf}}/\mathrm{dt}$ at regions demarcated in C vs [PIP2] fit to the Michaelis–Menten equation, Eq. **2**. R^2^ = 0.993. (*E*) Direct fit (shown as red curve) of the normalized current decay with $V_{\max}$, $K_{M}$, and *C* as free parameters (Eq. **4**). The gray dashed line denotes where the fit starts, which excludes an initial equilibration period. R^2^ = 0.975.

The measured current decays can be converted to PIP2 decays using the PIP2 concentration dependence of the channel, which we determined using titration experiments. Bilayers were formed with varying concentrations of long chain PIP2 from 0.1 to 4.0 mol% , GIRK-containing vesicles were fused, the current was measured, and water-soluble C8PIP2 (32 μM) was added to the Cis chamber to activate the channels maximally (*SI Appendix*, Fig. S1 *B* and *C*) (31). The measured current was normalized to the maximally activated current, $I_{max}$ , for each PIP2 concentration and fit to a modified Hill equation, Eq. **1**, to determine values A*, k*, and *r* (Fig. 2*A*):

[1] $$\frac{I}{I_{\max}}=A\frac{{\left[PIP2\right]}^{r}}{k^{r}+{\left[PIP2\right]}^{r}}.$$

Eq. **1** is an empirical function whose utility is to convert GIRK current into PIP2 concentration. In subsequent experiments with *PLCβ*3, bilayers initially contain 1.0 mol% PIP2 , which corresponds to ~30% of the maximal current (Fig. 2*A*).

The *PLCβ*3*/Gβγ*-dependent current decays were corrected by subtracting a constant current value representing nonspecific leak, then normalized to the starting PIP2 concentration (1.0 mol% ), and converted to PIP2 concentration decays using Eq. **1** with the predetermined values for k , A , and r (Fig. 2*B*). After an approximately 2 s delay associated with mixing of *PLCβ*3, PIP2 decays contained two components: an initial, approximately linear component followed by a slower, approximately exponential component (Fig. 2*C*). The linear component is consistent with *PLCβ*3 catalysis occurring as a 0th order reaction, where the catalytic rate is independent of the PIP2 concentration, suggesting that at our starting concentration (1.0 mol% PIP2 ), the active site of *PLCβ*3 is nearly fully occupied by substrate ( PIP2 ). The second, exponential, component is consistent with the PIP2 concentration becoming limiting to catalysis, a first-order reaction, as the decay progresses and the concentration of PIP2 decreases. In the example shown, for illustrative purpose, we estimated the rate within six intervals along the decay curve, demarcated with different colored circles (Fig. 2*C*), by measuring the slope to approximate $\frac{d[PIP2]}{\mathrm{dt}}$ within each interval, and then plotted the slope’s absolute value against the average PIP2 concentration for the corresponding interval (Fig. 2*D*). A Michaelis–Menten equation (Eq. **2**, below) fit the data points with R^2^ ~ 0.99, indicating that *PLCβ*3 catalytic activity can be described by this kinetic rate equation (Fig. 2*D*).

The graphical procedure described above and in Fig. 2 *C* and *D* was used as an example to place the PIP2 hydrolysis data into a familiar form of rate as a function of substrate concentration. For processing all data, we took a more direct approach to analyze the time-dependent decays within the Michaelis–Menten framework. Expressing the Michaelis–Menten rate equation as

[2] $$\frac{d[PIP2]}{\mathrm{dt}}=-\;\frac{V_{\max}\;\left[PIP2\right]}{K_{M}+\left[PIP2\right]},$$

and integrating from t = 0, we obtain for the PIP2 concentration as a function of time

**Fig. 3.**
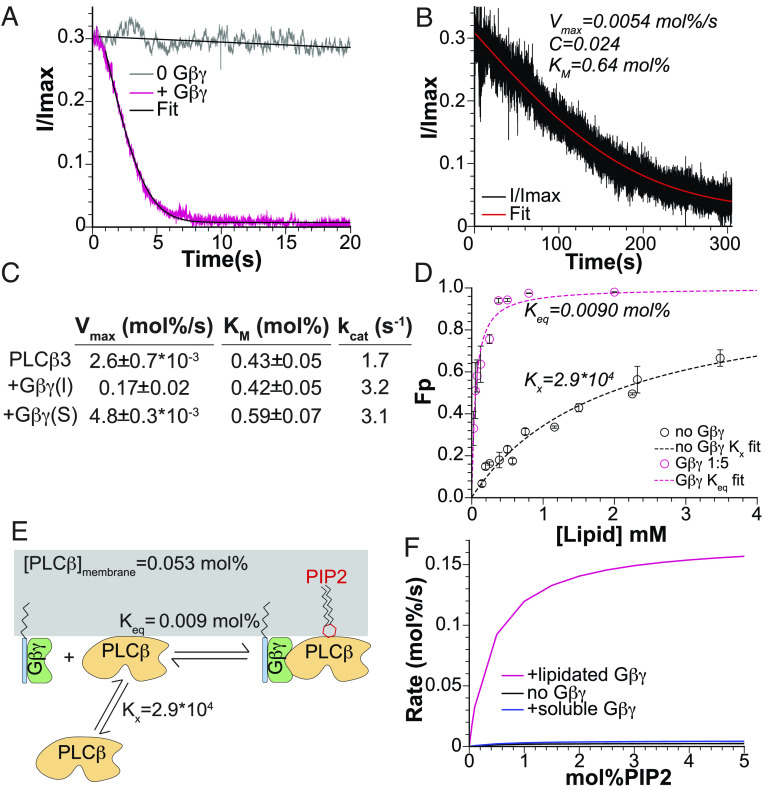
Gβγ activates *PLCβ*3 by increasing its concentration at the membrane. (*A*) Comparison of normalized current decay in the presence (pink) and absence (gray) of lipidated Gβγ fit to Eq. **4** (black curves). Results from the fit without Gβγ : $V_{\max}$ =0.0023 ± 0.6E-6 $\frac{mol\%}{s}$ , C = −0.03 ± 0.0004, $K_{M}$ = 0.43 ± 0.0008 mol% , R^2^ = 0.992. With Gβγ : $V_{\max}$ = 0.22 ± 0.0001 $\frac{mol\%}{s}$ , C = 0.0074 ± 5E-5, $K_{M}$ = 0.37 ± 0.0006 mol% . (*B*) Normalized current decay in the presence of 1 μM  soluble Gβγ fit to Eq. **4** (red curve). R^2^ = 0.955. (*C*) Comparison of $V_{\max}$ , $K_{M}$ , and $k_{\mathrm{cat}}$ for *PLCβ*3 alone, with lipidated Gβγ  ( Gβγ (l)) and with soluble Gβγ (Gβγ (s)). (*D*) Membrane partitioning curve for *PLCβ*3 alone (black) or in the presence of lipidated Gβγ (pink) for 2DOPE:1POPC:1POPS LUVs with Fraction Partitioned ( $F_{p}$ ) on the *Y* axis. Data for 0 Gβγ were fit to Eq. **6** for $K_{x}$ (dashed black curve) and data for +Gβγ were fit to Eq. **7** to determine $K_{\mathrm{eq}}$ (39). Error bars are range of mean from two experiments for each lipid concentration. R^2^ = 0.96 in the absence of Gβγ and R^2^ = 0.95 in the presence of Gβγ . (*E*) Cartoon representation of *PLCβ*3 activation by Gβγ through membrane recruitment. Gβγ significantly increases the membrane association of *PLCβ*, and accordingly [*PLCβ*]_membrane_, which amplifies PIP2 hydrolysis. [*PLCβ*]_membrane_ was calculated from Eq. **8** using [*PLCβ*_w_]=5.3E-8 *mol%*, [*Gβγ*]=[*Gtot*]=0.34 *mol%*, and *K_x_* and *K_eq_*, which were determined through the fits in panel *D*. (*F*) Calculated Michaelis–Menten curves (from Eq. **2**) for *PLCβ*3 alone (black), in the presence of 1 μM soluble Gβγ (blue) or in the presence of lipidated Gβγ (pink) using the values for $K_{M}$ and $V_{\max}$ determined from our fits.

[3] $$\begin{array}{c} \left[PIP2\left(t\right)\right]=K_{M}ProductLog\;\frac{e^{\frac{\left(\left[PIP2\left(0\right)\right]-tV_{\max}\right)}{K_{M}}}\left[PIP2\left(0\right)\right]}{K_{M}}, \end{array}$$

where $\left[PIP2\left(0\right)\right]$ is the PIP2 concentration at t = 0 and *K_M_* and *V_max_* are the Michaelis–Menten parameters. Eq. **3** derived here contains a well-known function called the Lambert W function or ProductLog function (38). It describes for the PIP2 concentration an initially linear decay followed by an exponential decay. Substituting Eq. **3** into Eq. **1**, we obtain an expression for GIRK current decay as a function of time due to PIP2 hydrolysis,

[4] $$\begin{array}{c} \frac{I}{I_{max}}=C+\;A\;\frac{[PIP2(t){]}^{r}}{k^{r}+[PIP2(t){]}^{r}}, \end{array}$$

which permits direct fitting of the normalized current decays to estimate *V_max_* and *K_M_* (Fig. 2*E*).  $[PIP2\left(t\right)]$ in **Eq. 4** is given by **Eq. 3**, and a third free parameter, C , accounts for the level of background leak in bilayer experiments; this is visible as the small residual current (typically ≤ 5% of the GIRK current) at long times in Figs. 2*E* and 3*A*. $\left[PIP2\left(0\right)\right],$ the initial PIP2 concentration, is specified by the bilayer composition and A , k, and r are predetermined through the fit of Eq. **1** to the data shown in Fig. 2*A*. Eq. **4** fits the current decay data accurately after ~2 s (Fig. 2*E*) and yields consistent results for $V_{\max}$ (0.17 ± 0.02 mol%/sec ) and $K_{M}$ (0.42±0.05 mol% ) across repeated experiments (Fig. 3*C*).

### The Role of *Gβγ* in the Function of *PLCβ*3.

In the experiments described above, Gβγ was added to the planar lipid bilayers by equilibrating lipid vesicles containing Gβγ with the bilayer surface prior to the application of *PLCβ*3. When Gβγ is not added to the bilayer, *PLCβ*3 produces a much slower current decay, as shown (Fig. 3*A* and *SI Appendix*, Fig. S1 *D* and *E*). Similarly, in the presence of 1 *µM* aqueous-soluble Gβγ without a lipid anchor, which does not partition onto the membrane surface (31), *PLCβ*3 catalyzed current decay is also slow (Fig. 3 *B* and *C*). Seven experiments were carried out in the absence of Gβγ and the rmsd between the current decay curves and Eq. **4** were minimized to yield $V_{\max}$ (0.0026 ± 0.0007 mol%/sec ) and $K_{M}$ (0.43 ± 0.05 mol% ) (Fig. 3*C*). Thus, Gβγ in the membrane increases $V_{\max}$ ~65-fold without affecting $K_{M}$ (Fig. 3*C*).

Because *PLCβ*3 is soluble in aqueous solution but must localize to the membrane surface to catalyze PIP2 hydrolysis, we next examined whether Gβγ in the membrane influences *PLCβ*3 membrane localization. As detailed by White and colleagues, protein association with membranes cannot be considered as a simple binding equilibrium due to the fluid nature of the membrane without discrete binding sites (39). Instead, membrane association must be treated as a partitioning process between two immiscible solvents, the membrane and the aqueous solution. The equilibrium partition coefficient, *K_x_,* is the ratio of the mole fraction of *PLCβ*3 in the membrane (subscript *m*) to that in aqueous solution (subscript *w*) (39),

[5] $$K_{x}=\frac{\left[{PLC\beta 3}_{m}\right]}{\left[{PLC\beta 3}_{w}\right]}.$$

To determine the value of $K_{x}$ , detergent-free liposomes were reconstituted using 2DOPE:1POPC:1POPS lipids to match the lipid composition of the bilayer experiments, and H^+^ NMR was used to measure the lipid concentration at the end of the detergent removal process (*SI Appendix*, Fig. S2*A*). Large unilamellar vesicles (LUVs) were prepared from the reconstituted liposomes using freeze–thaw cycles and extrusion through a 200 nm membrane. The LUVs were incubated with *PLCβ*3 and pelleted using ultracentrifugation to separate the membrane-bound and aqueous protein fractions. This method allows direct measurement of both the bound and free protein using fluorescently labeled *PLCβ*3, which facilitates determining the partition coefficient from each experiment individually (39). The membrane-associated fraction of *PLCβ*3, fraction partitioned ( $F_{p}$), is

[6] $$\begin{array}{c} F_{p}=\frac{\left[PLC\beta 3_{m}\right]\;{\left[L\right]}_{\mathrm{molar}}}{\left[PLC\beta 3_{m}\right]\;{\left[L\right]}_{\mathrm{molar}}+\left[PLC\beta 3_{w}\right]\;{\left[W\right]}_{\mathrm{molar}}}\\ \;\;=\frac{K_{x}\;{\left[L\right]}_{\mathrm{molar}}}{K_{x}\;{\left[L\right]}_{\mathrm{molar}}+{\left[W\right]}_{\mathrm{molar}}}. \end{array}$$

${\left[W\right]}_{\mathrm{molar}}$ , the molar concentration of water, is ~55 M and ${\left[L\right]}_{\mathrm{molar}}$ , the molar concentration of lipid, is set for each experiment using a stock measured by NMR. Thus, Eq. **6** is a function of the single free parameter, $K_{x}$ , which we determine by fitting Eq. **6** to the partitioning data, yielding $K_{x}$ ~$2.9\;\cdot \;10^{4}$ (Fig. 3*D*, black curve). Partitioning experiments carried out with unlabeled *PLCβ*3 quantified using sodium dodecyl sulfate–polyacrylamide gel electrophoresis (SDS-PAGE) analysis yielded a similar value of $K_{x}$ ( $∼4\cdot 10^{4}$ ) (*SI Appendix*, Fig. S2 *B* and *C*), confirming that the fluorescent label does not alter the partitioning behavior of *PLCβ*3.

LUVs with the same lipid composition were also prepared containing Gβγ , which is exclusively membrane bound, at a protein to lipid ratio of 1:5 (wt:wt), corresponding to 0.34 mol% , to match the concentration of Gβγ in vesicles equilibrated with planar lipid bilayers in the kinetic experiments. At this concentration of Gβγ , we observe that *PLCβ*3 binds to vesicles much more readily than in the absence of Gβγ (Fig. 3*D*). This observation is explicable if, when *PLCβ*3 partitions onto the membrane surface, it binds to Gβγ . Writing the binding reaction on the membrane surface as ${PLC\beta 3}_{m}+G\beta \gamma ⇌PLC\beta 3\cdot G\beta \gamma$ , we have $K_{\mathrm{eq}}=$ $\frac{\left[PLC\beta 3_{m}][G\beta \gamma \right]}{\left[PLC\beta 3\cdot G\beta \gamma \right]}$ (Fig. 3*E*). (Note that subscript m indicates PLCβ3 on the membrane. Since Gβγ only resides on the membrane, a subscript is not used for $\left[G\beta \gamma \right]\;and\;\left[PLC\beta 3\cdot G\beta \gamma \right]$ ). When equilibrium is reached, the membrane surface will contain a quantity of PLCβ3 in the membrane that is not bound to Gβγ , set by $K_{x}$ and the aqueous solution concentration of PLCβ3 , as well as a quantity of PLCβ3 in the membrane that is bound to Gβγ (i.e., PLCβ3⋅Gβγ) , set by the membrane concentrations of PLCβ3 , Gβγ and $K_{\mathrm{eq}}$ . Therefore, in the presence of a total quantity of Gβγ on the membrane, $\left[Gtot\right]=\left[G\beta \gamma \right]$ $+\left[G\beta \gamma \cdot PLC\beta 3\right]$ , the fraction of PLCβ3 on the membrane surface, unbound plus bound to Gβγ , is given by (SI Appendix 2)

[7] $$\begin{array}{c} F_{p}\left(\;+\;G\beta \gamma \right)=\frac{K_{x}\;{\left[L\right]\;}_{\mathrm{molar}}\left(f\left(x\right)+\;2\;\left[Gtot\right]{\left[W\right]}_{\mathrm{molar}}\right)}{{(K}_{x}\;{\left[L\right]}_{\mathrm{molar}}+{\left[W\right]}_{\mathrm{molar}})\;f\left(x\right)}, \end{array}$$

with $\begin{array}{c} f\left(x\right)=p+q+x+{\left\{4\;q\;x+{\left(p-q+x\right)}^{2}\right\}}^{1/2} \end{array}$ , where $p=K_{x}$${\;\left[L\right]}_{\mathrm{molar}}{\;[G}_{\mathrm{tot}}]$ , $q=K_{x}\;[{\mathrm{PLC}}_{\mathrm{tot}}]\;{\;}_{\mathrm{molar}}$ , and $x=K_{\mathrm{eq}}\;({\left[W\right]}_{\mathrm{molar}}+$$K_{x}{\;\left[L\right]}_{\mathrm{molar}})$ . Because ${\left[{\mathrm{PLC}}_{\mathrm{tot}}\right]}_{\mathrm{molar}}$ (the molar concentration of PLCβ3 ( ${PLC\beta 3}_{w}$ and ${PLC\beta 3}_{m}$ ) plus PLCβ3⋅Gβγ ), ${\left[L\right]}_{\mathrm{molar}}$ and ${\left[W\right]}_{\mathrm{molar}}$ (molar concentrations of lipid and water) and [Gtot] ( mf Gβγ plus PLCβ3⋅Gβγ in the membrane) are established in the experimental setup, and $K_{x}$ is determined through partition measurements in the absence of Gβγ (Fig. 3*D*), the right-hand side of Eq. **7** contains a single free parameter, $K_{\mathrm{eq}}$ , for the binding of PLCβ3 to Gβγ on the lipid membrane surface. The red dashed curve in Fig. 3*D* corresponds to $K_{\mathrm{eq}}$ = 0.0090 mol% . It may seem at first surprising that the series of partitioning experiments in the presence of Gβγ , with knowledge of $K_{x}$ for PLCβ3 in the absence of Gβγ , uncovers the equilibrium reaction between PLCβ3 and Gβγ on the membrane surface. Nevertheless, the binding reaction is discernable by this approach, and the inescapable conclusion is that Gβγ concentrates PLCβ3 on the membrane surface (Fig. 3*E*).

The PLCβ3 -concentrating effect of Gβγ has obvious implications for interpreting the kinetic data reported above, which show that Gβγ increases $V_{\max}$ by a factor ~65, without affecting $K_{M}$ very much (Fig. 3*C*). From Eq. **2**, $V_{\max}$ is the asymptotic rate of PIP2 hydrolysis when [ PIP2 ] far exceeds $K_{M}$ . In this limit, the maximum rate of hydrolysis, $V_{\max}$ , is given by the total membrane concentration of PLCβ3 times $k_{\mathrm{cat}}$ , the turnover rate of a PLCβ3⋅PIP2 complex. In the bilayer chamber used for the kinetic experiments, the volume of the aqueous solution is so large compared to the small area of the lipid bilayer that surface binding does not significantly alter $\left[{PLC\beta 3}_{w}\right]$ . Under this condition, we have

[8] $$\begin{array}{c} V_{\max}=K_{x}\;\left[{PLC\beta 3}_{w}\right]\left(1+\frac{\left[G_{\mathrm{tot}}\right]}{K_{\mathrm{eq}}+K_{x\;}\left[{PLC\beta 3}_{w}\right]}\right)k_{\mathrm{cat}}, \end{array}$$

where $K_{x}\;\left[{PLC\beta 3}_{w}\right]$ is the membrane concentration of PLCβ3 in the absence of Gβγ ( ${[G}_{\mathrm{tot}}]=0$ ) and $K_{x}\;\left[{PLC\beta 3}_{w}\right]$ $\left(1+\frac{\left[G_{\mathrm{tot}}\right]}{K_{\mathrm{eq}}+K_{x\;}\left[{PLC\beta 3}_{w}\right]}\right)$ is the membrane concentration in its presence ( ${[G}_{\mathrm{tot}}]>0$ ). Thus, the term $\left(1+\frac{\left[G_{\mathrm{tot}}\right]}{K_{\mathrm{eq}}+K_{x\;}\left[{PLC\beta 3}_{w}\right]}\right)$ is a multiplier giving the fold-increase in total membrane PLCβ3 concentration due to the presence of Gβγ at concentration ${[G}_{\mathrm{tot}}]$ . When the known quantities are entered for our experimental conditions, this factor is ~33. In the kinetic experiments, we observed a 65-fold increase in $V_{\max}$ in the presence of Gβγ . Eq. **8** predicts a 33-fold increase through Gβγ's ability to increase the local concentration of PLCβ3 on the membrane surface. A mere two-fold increase in $k_{\mathrm{cat}}$ produced by Gβγ binding to PLCβ3 would account for the full enhancement of $V_{\max}$ in the kinetic experiments (Fig. 3*C*). The important conclusion is that most of the increase in $V_{\max}$ (within a factor of ~2) is explained by the ability of Gβγ to concentrate PLCβ3 on the membrane surface. Indeed, it seems very possible that the ~two-fold shortfall is accountable by the ability of Gβγ to orient PLCβ3 , in addition to concentrating it. Using a conventional Michaelis–Menten plot, with the $V_{\max}$ and $K_{M}$ values derived experimentally, we observe that at concentrations in our assay, Gβγ essentially switches the PLCβ3 enzyme on (Fig. 3*F*), and this effect is due largely to the ability of Gβγ to concentrate PLCβ3 on the membrane surface.

In summary, the kinetic studies show that PLCβ3 catalyzes PIP2 hydrolysis with a substrate concentration dependence like that of a Michaelis–Menten enzyme (Fig. 2 *C*–*E*). We note that $K_{M}$ corresponds to the mid-range of known PIP2 concentrations in cell membranes (Figs. 2*D* and 3*F*) (40, 41). PLCβ3 aqueous-membrane partition studies show that Gβγ concentrates PLCβ3 on the membrane surface, enough to account for most of the effect on $V_{\max}$ (Fig. 3 *C* and *D*). To a smaller extent (~two-fold), Gβγ augments $V_{\max}$ through $k_{\mathrm{cat}}$ (Fig. 3*C*). Next, we evaluate the structural underpinnings of these functional properties.

### Structural Studies of *PLCβ*3 in Aqueous Solution by Cryo-EM.

**Fig. 4.**
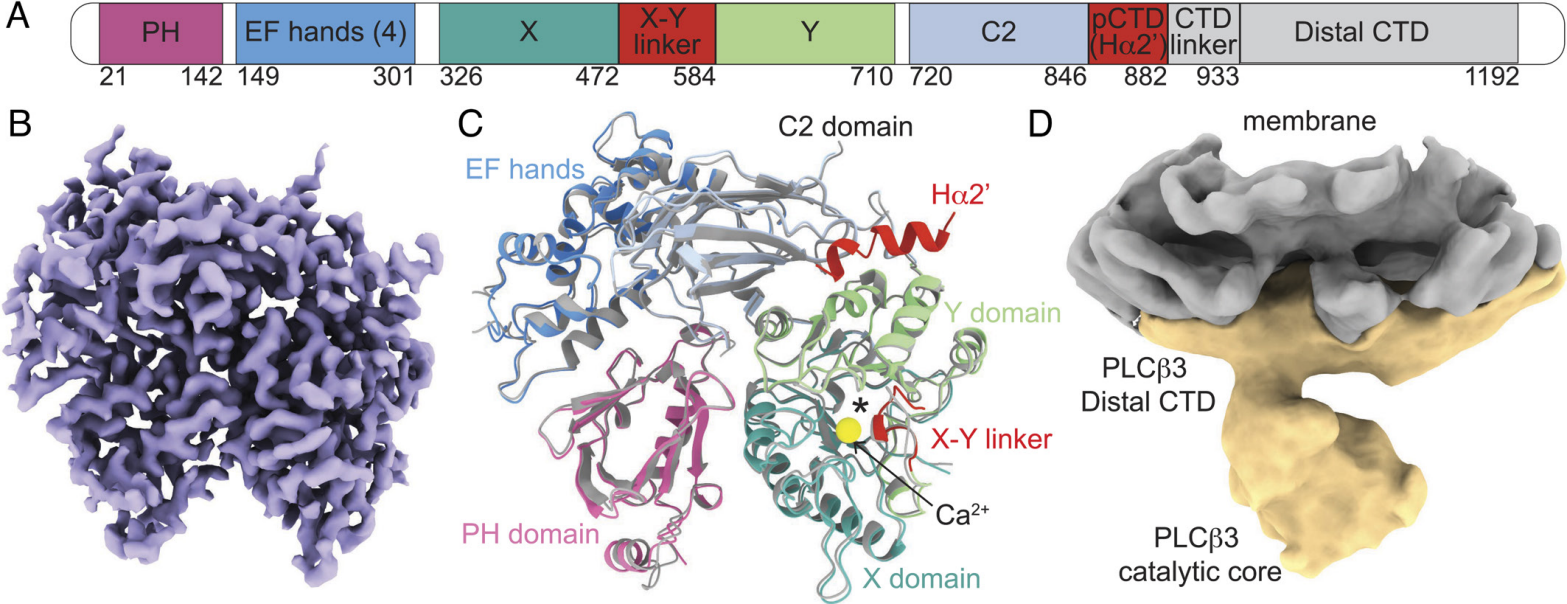
Structures of *PLCβ*3 in solution and on vesicles without Gβγ . (*A*) primary structure arrangement of *PLCβ* enzymes. Sections are colored by domain as in *C*. Domains in gray (CTD linker and Distal CTD) are not observed in our structures. pCTD is proximal CTD, of which only the Hα2′ is resolved. (*B*) Sharpened, masked map of *PLCβ*3 catalytic core obtained from a sample in solution without membranes or detergent. (*C*) Structural alignment of the catalytic core of *PLCβ*3 from the crystal structure of the full-length protein bound to ${G\alpha }_{q}$ [colored in gray, PDBID: 4GNK, (19)] and the structure determined using cryo-EM without membranes (colored by domain). Cα rmsd is 0.6 Å. Calcium ion from the cryo-EM structure is shown as a yellow sphere, and the active site is denoted with an asterisk. The PH domain is pink, the EF hand repeats are blue, the C2 domain is light blue, the Y domain is green, the X domain is teal, and the X–Y linker and the Hα2’ are red. (*D*) Unsharpened reconstruction of *PLCβ*3 bound to lipid vesicles containing 2DOPE:1POPC:1POPS. *PLCβ*3 is colored in yellow and the membrane is colored in gray.

We next determined the structure of *PLCβ*3 in aqueous solution using cryo-EM. The structure, consisting of the *PLCβ*3 catalytic core at 3.6 Å resolution, contained the PH domain, EF hands, X and Y domains, the C-terminal part of the X-Y linker, the C2 domain, and the active site with a Ca^2+^ ion bound (Fig. 4 *A* and *B* and *SI Appendix*, Fig. S3 and Table S1). The autoinhibitory Hα2′ element in the proximal CTD was also resolved, bound to the catalytic core between the Y domain and the C2 domain, as proposed by Lyon and colleagues (Fig. 4 *A* and *B*) (16, 21) but not the distal CTD. We also obtained several low-resolution reconstructions with varying levels of density corresponding to the catalytic core and distal CTD with differing arrangements between the two domains (*SI Appendix*, Fig. S3*F*). This observation suggests that the distal CTD is disordered rather than proteolyzed in our final reconstruction and that the two domains are flexible with respect to each other, as previously proposed (19). The catalytic core resolved by cryo-EM is very similar to the crystal structure with a Cα rmsd of 0.6 Å if the Hα2′ helix is excluded (Fig. 4*C*). We note that, as in the crystal structure, the autoinhibitory X–Y linker occludes the active site (Fig. 4*C*). We attempted to determine a structure of *PLCβ*3 in complex with Gβγ in solution, in the presence or absence of detergent, without success. Furthermore, we were unable to detect the formation of a complex in solution by size-exclusion chromatography (*SI Appendix*, Fig. S3*G*).

### Structural Studies of *PLCβ*3 Associated with Liposomes.

We next determined the structure of *PLCβ*3 bound to liposomes consisting of 2DOPE:1POPC:1POPS. PIP2  was omitted from these samples because it would have been degraded by *PLCβ*3 prior to grid preparation. We obtained a low-resolution reconstruction with the distal CTD associated with the membrane and the catalytic core located away from the membrane surface (Fig. 4*C* and *SI Appendix*, Fig. S4 and Table S1). Although the map was low resolution, previously determined structures fit into the density for each domain and all reconstructions showed the same orientation of the protein on the membrane surface (*SI Appendix*, Fig. S4). The interaction of the distal CTD with the membrane is consistent with previous reports of its involvement in membrane association (3). The position of the catalytic core indicates that significant rearrangements of *PLCβ*3 with respect to the membrane must be involved in activation because the active site is too far from the membrane to access PIP2 . Activating rearrangements could be mediated by interactions of lipid-anchored G proteins with the *PLCβ*3 catalytic core.

### The *PLCβ3 · Gβγ* Complex on Liposomes Reveals Two *Gβγ* Binding Sites.

**Fig. 5.**
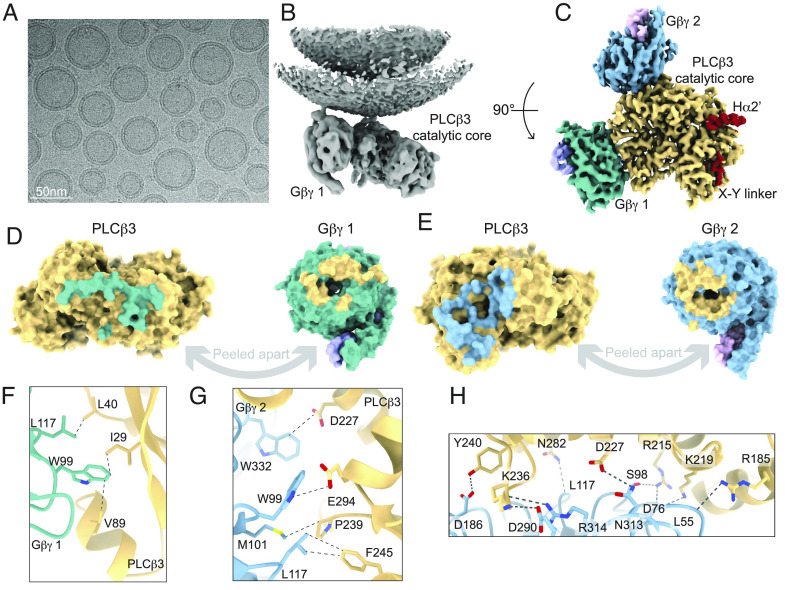
*PLCβ*3 · *Gβγ* complex on lipid vesicles and Gβγ interfaces. (*A*) Example micrograph showing lipid vesicles with protein complexes. (*B*) Unsharpened map from nonuniform refinement showing the *PLCβ*3 · *Gβγ* complex on the vesicle surface. Both the inner and outer leaflets of the vesicle are shown. (*C*) Sharpened, masked map of the catalytic core of *PLCβ*3 in complex with two *Gβγs* on lipid vesicles containing 2DOPE:1POPC:1POPS. *PLCβ*3 is yellow, Gβ 1 is dark teal, Gγ 1 is light purple, Gβ 2 is light blue, and Gγ 2 is light pink. The autoinhibitory elements Hα2′ and the X–Y linker are colored in red. Coloring is the same throughout. D-E: Surface representation of the *PLCβ*-*Gβγ* 1 (*D*) or *PLCβ*-*Gβγ* 2 (*E*) interfaces peeled apart to show extensive interactions. Residues on *PLCβ*3 that interact with Gβγ 1 or 2 are colored according to the corresponding Gβ coloring and residues on the Gβ s that interact with *PLCβ*3 are colored in yellow. Interface residues were determined using the ChimeraX interface feature using a buried surface area cutoff of 15 Å^2^. (*F* and *G*) Interactions of residues on Gβ that have been shown to be important for *PLCβ* activation with residues from *PLCβ*3 in the *PLCβ*-*Gβγ* 1 interface (*F*) or the *PLCβ*-*Gβγ* 2 interface (*G*) (43). All labeled interactions are < ~4 Å. Interacting residues are shown as sticks and colored by heteroatom. Interactions are denoted by black dashed lines. (*H*) Extensive hydrogen bond network in the *PLCβ*-*Gβγ* 2 interface including both sidechain and backbone interactions. All labeled hydrogen bonds are between ~2.3 and ~3.8 Å. Interacting residues are shown as sticks and colored by heteroatom. Interactions are denoted by black dashed lines.

We reconstituted Gβγ into liposomes consisting of 2DOPE:1POPC:1POPS at a protein to lipid ratio of 1:15 (wt:wt) and incubated the liposomes with purified *PLCβ*3 prior to grid preparation. We determined the structure of the *PLCβ*3· *Gβγ* complex to 3.5 Å and observed two *Gβγs* bound to the catalytic core of *PLCβ*3 (Fig. 5 *A*–*C* and *SI Appendix*, Fig. S5 and Table S1). The distal CTD is not resolved in our reconstructions, suggesting that it might adopt many different orientations on the plane of the membrane relative to the catalytic core, in agreement with previous studies showing that heterogeneity in the distal CTD increases upon Gβγ binding (42). The catalytic core is very similar to our cryo-EM structure without membranes, with a Cα rmsd of 0.7 Å. Only small rearrangements occur at the Gβγ binding sites (*SI Appendix*, Fig. S6*A*). Both autoinhibitory elements, the Hα2' and the X–Y linker, are engaged with the catalytic core (Fig. 5*C*) consistent with previous proposals that Gβγ does not play a role in relieving this autoinhibition (15, 16, 21).

One Gβγ is bound to the PH domain and the first EF hand, referred to as Gβγ 1, and the other is bound to the remaining EF hands, referred to as Gβγ 2 (Fig. 5*C* and *SI Appendix*, Fig. S6 *A* and *B*). Both interfaces are extensive, with the Gβγ 1 interface burying ~800 Å^2^ and involving 34 residues, (16 from *PLCβ*3 and 18 from Gβγ ) and the Gβγ 2 interface burying ~1,100 Å^2^ and involving 44 residues (21 from *PLCβ*3 and 23 from Gβγ ) (Fig. 5 *D* and *E* and *SI Appendix*, Fig. S6*B* and Table S2). The Gβγ 1 interface is mostly composed of hydrophobic interactions, with three hydrogen bonds (Fig. 5*F* and *SI Appendix*, Fig. S6*C*), whereas the Gβγ 2 interface is mostly composed of electrostatic interactions, including 10 hydrogen bonds spanning the length of the interface (Fig. 5 *G* and *H*). Both interfaces involve the same region of Gβγ that interacts with Gα and several residues on Gβ shown to be important for *PLCβ* activation are involved (43) (Fig. 5 *E* and *F*). Specifically, L117 and W99 on Gβ 1 form hydrophobic interactions with L40, I29, and V89 on *PLCβ*3 (Fig. 5*F* and *SI Appendix*, Table S2) (43). On Gβ 2, W99 forms a hydrogen bond with E294 on *PLCβ*3, W332 forms an anion-edge interaction with D227, M101 and L117 form hydrophobic interactions with P239 and F245 on *PLCβ*3, and D186 forms a hydrogen bond with Y240 on *PLCβ*3 (Fig. 5 *G* and *H* and *SI Appendix*, Table S2) (43).

We also determined the structure of the *PLCβ*3 · *Gβγ* complex using lipid nanodiscs. We reconstituted Gβγ into nanodiscs formed using the MSP2N2 scaffold protein (44) and 2DOPE:1POPC:1POPS lipids and incubated them with purified *PLCβ*3 prior to grid preparation. We observed only reconstructions with two *Gβγs* bound and determined the structure of the complex to 3.3 Å (*SI Appendix*, Fig. S7). The two Gβγs are bound in the same locations as was observed in liposomes with comparable interfaces (*SI Appendix*, Fig. S6*D*). A model for this structure aligns well to the model built using the lipid vesicle reconstruction with a Cα rmsd of 0.8 Å for all proteins (*SI Appendix*, Fig. S6*D*). These structures suggest that the *PLCβ*3 · *Gβγ* complex depends on a membrane environment as we were unable to form a stable complex in solution with or without detergent, which highlights the importance of the membrane in *Gβγ*-dependent activation of *PLCβ*3.

### *Gβγ* Mediates Membrane Association and Orientation of the *PLCβ*3 Catalytic Core.

**Fig. 6.**
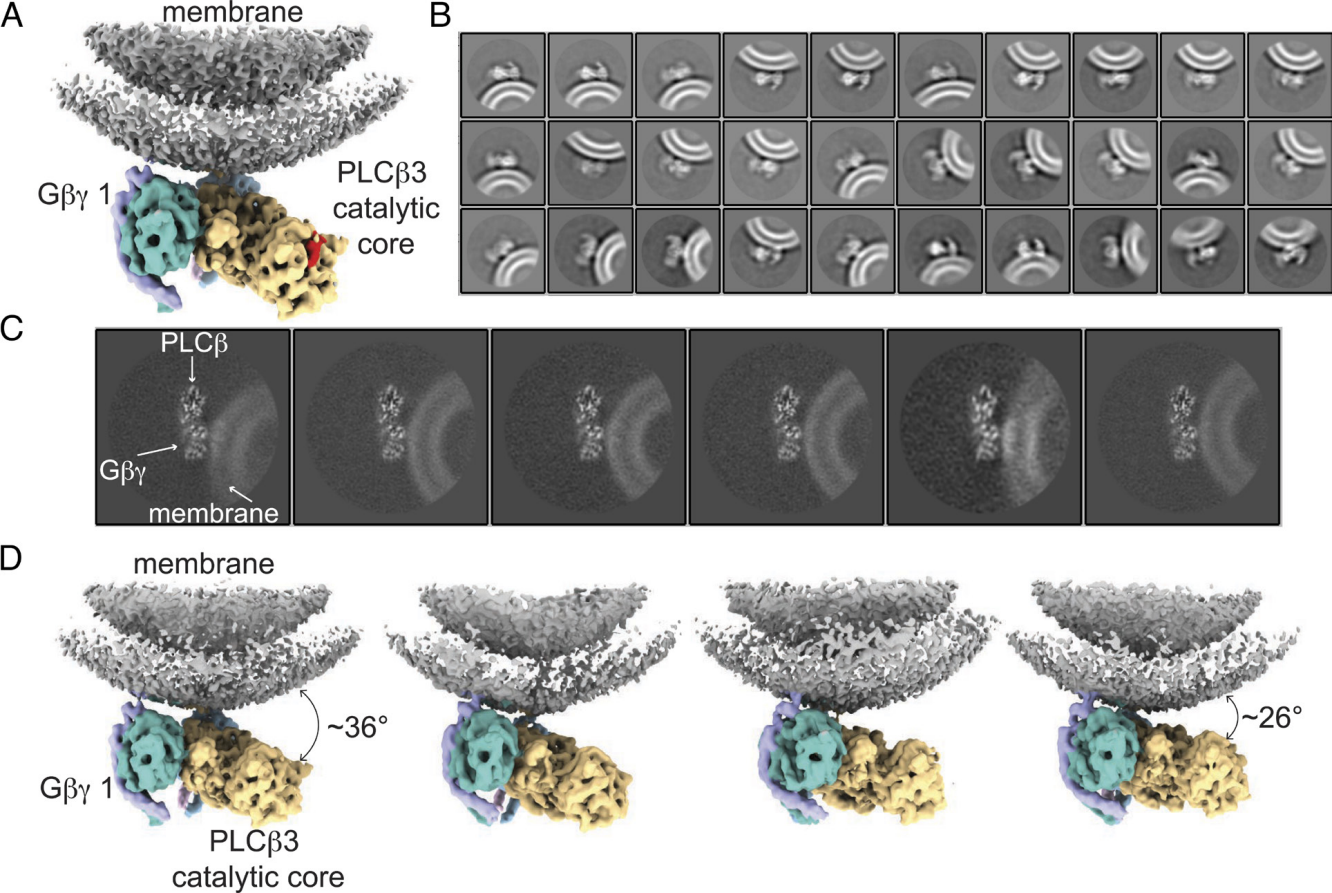
Tilting of the *PLCβ*3 · *Gβγ* complex with respect to the membrane. (*A*) Consensus unmasked refinement with density for the *PLCβ*3 · *Gβγ* complex and the membrane colored by protein. The membrane is gray, *PLCβ*3 is yellow, Gβ 1 is dark cyan, and Gγ 1 is light purple. The X–Y linker is colored red to highlight the active site. (*B*) 2D class averages of the final subset of particles determined without alignment showing side views of the complex on the membrane. Different membrane curvatures and positions of the complex with respect to the membrane are demonstrated. (*C*) 2D projections of 3D classes of the *PLCβ*3 · *Gβγ* complex on the membrane. (*D*) 3D reconstructions of four 3D classes with different positions of the complex on the membrane arranged by degree of tilting with the most tilted on the left and least tilted on the right.

Unmasked refinement of our final subset of particles from the liposome structure yielded a 3.8 Å reconstruction showing the *PLCβ*3 · *Gβγ* assembly and density from the membrane (Figs. 5*B* and 6*A*). The two *Gβγs* and the region of *PLCβ*3 between them are closely associated with the membrane and the remainder of the catalytic core, including the active site, tilts away from the membrane (Fig. 6*A*). Despite the tilting, the structure reveals significant rearrangement of the catalytic core with respect to the membrane compared to its position in the absence of Gβγ , where it was separated from the membrane surface by a larger distance (Fig. 6*A*).

Additional 2D and 3D classification without alignment revealed heterogeneity in the position of the *PLCβ*3 · *Gβγ* assembly with respect to the membrane (Fig. 6 *B*–*D*). 2D classes show large variation in the orientation of the catalytic core with respect to the membrane surface, with some classes showing the entire catalytic core engaged with the membrane (Fig. 6*B*). The 2D classes also reveal differences in membrane curvature originating from differences in liposome size, which do not seem to be correlated with the degree of membrane tilting (Fig. 6*B*). 3D classification revealed four reconstructions capturing different degrees of tilting of the catalytic core ranging from ~26° to ~36° (Fig. 6*D*). We note that in a locally planar membrane, as opposed to a curved vesicle membrane, the active site would be nearer the membrane surface in all classes, but the variability in orientation would presumably still exist. The protein components of these reconstructions are like in the original reconstruction, with no internal conformational changes, indicating that the whole complex tilts on the membrane as a rigid body.

**Fig. 7.**
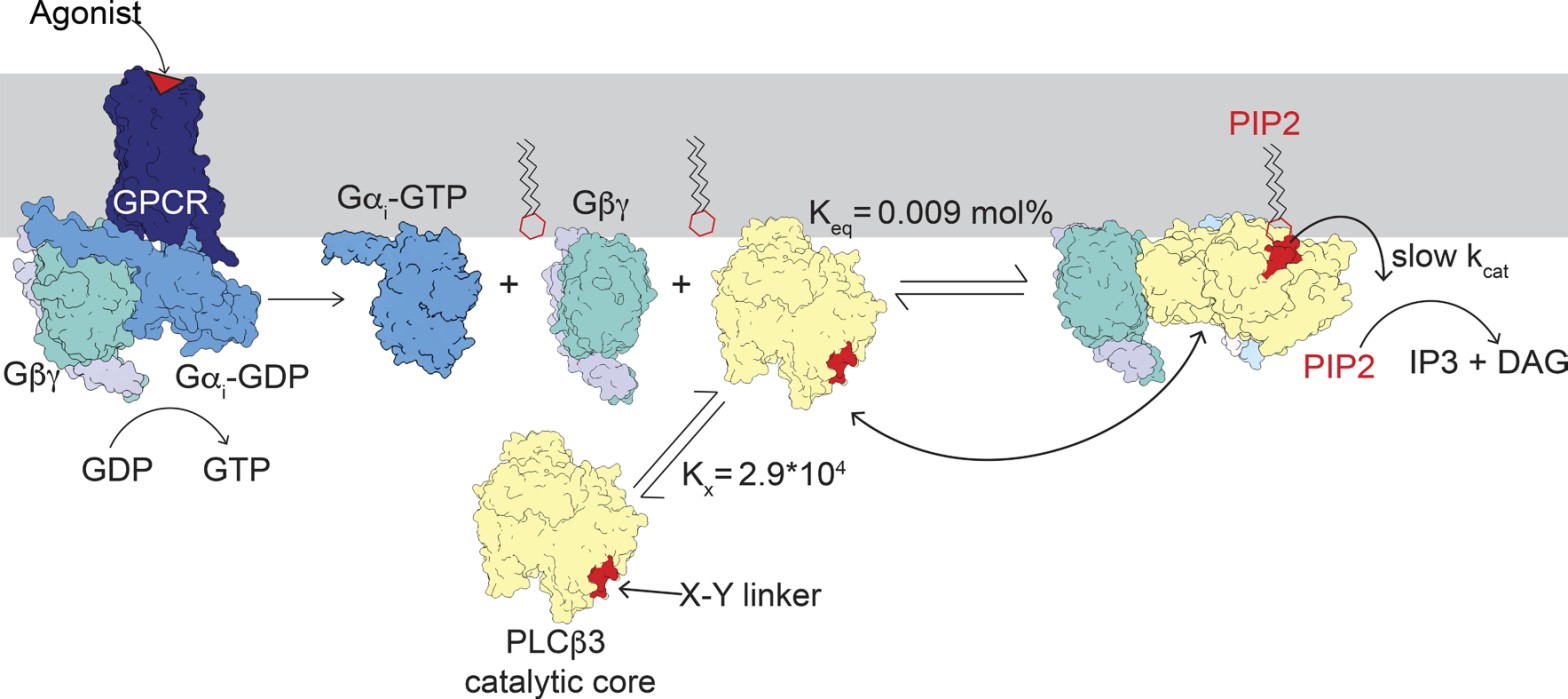
Gβγ activates PLCβ by increasing its concentration at the membrane and orienting the catalytic core to engage PIP2 . Upon activation of a ${G\alpha }_{i}$-coupled receptor, GTP is exchanged for GDP in the ${G\alpha }_{i}$ subunit and free Gβγ is released to bind *PLCβ,* which increases the concentration of *PLCβ* at the membrane and orients the active site for catalysis. The $k_{\mathrm{cat}}$ is limited by the X–Y linker (shown in red), which occludes the active site and is only transiently displaced from the active site to allow catalysis. The distal CTD of *PLCβ* was omitted for clarity.

The lack of conformational changes observed upon Gβγ binding and the catalytic core membrane association are consistent with our functional studies showing that activation by Gβγ is largely mediated by increasing membrane partitioning. Our structures suggest that the configuration of the two Gβγ binding sites maintains the catalytic core at the membrane and increases the probability of productive engagement with PIP2 , potentially mediated by orientation of the catalytic core observed in our reconstructions. Taken together, our kinetic, binding, and structural studies lead us to conclude that Gβγ activates PLCβ mainly by bringing it to the membrane and orienting the catalytic core so that the active site can access the PIP2-containing surface (Fig. 7).

## Discussion

This study aims to understand how a G protein, Gβγ , activates the PLCβ3 phospholipase enzyme. We developed and applied three new technical approaches to study this process. First, because kinetic analyses of PLCβ enzymes historically have been limited to relatively slow radioactivity-based or semiquantitative fluorescence assays, we have developed a new higher resolution assay using a modified, calibrated PIP2-dependent ion channel to provide a direct read out of membrane PIP2 concentration as a function of time. This assay is employed in a reconstituted system in which all components are defined with respect to composition and concentration. Second, we have used a membrane-water partition assay to study a surface equilibrium reaction between two proteins ( PLCβ3 and Gβγ ) on membranes. Third, we have determined structures of a protein complex ( PLCβ3 and Gβγ ) assembled on the surface of pure lipid vesicles. We also determined the structures using lipid nanodiscs; however, the lipid vesicles permitted structural analysis of the enzyme-G protein complex on lipid surfaces unperturbed by the scaffold proteins required to make nanodiscs. The membrane in our nanodisc reconstructions is poorly resolved and the complex appears to be associated at nonphysiological orientations; therefore, we cannot gain any information regarding the positioning of the complex on the membrane from those reconstructions.

We list our essential findings. 1) PLCβ3 catalyzes PIP2 hydrolysis in accordance with Michaelis–Menten enzyme kinetics. 2) Gβγ modifies $V_{\max}$ , leaving $K_{M}$ essentially unchanged. Under our experimental conditions, $V_{\max}$ increases ~65-fold. 3) Gβγ increases membrane partitioning of PLCβ3 , an effect accountable through equilibrium complex formation between Gβγ and PLCβ3 on the membrane surface. Under our experimental conditions, partitioning increases the membrane concentration of PLCβ3 ~33-fold. 4) The Gβγ -mediated increase in PLCβ3 partitioning can account for most of the increase in $V_{\max}$ , with a smaller, ~two-fold, effect on $k_{\mathrm{cat}}$ . Thus, Gβγ regulates PLCβ3 mainly by concentrating it on the membrane. 5) Two Gβγ proteins assemble to form a complex with PLCβ3 on vesicle surfaces. One Gβγ binds to the PH domain and one EF hand of PLCβ3 , while the other binds to the remaining EF hands. Both Gβγ orient their covalent lipid groups toward the membrane so that the PLCβ3 catalytic core is firmly anchored on the membrane surface. 6) The PLCβ3⋅Gβγ assembly holds the PLCβ3 catalytic core with its active site, as if on the end of a stylus, poised to sample the membrane surface. Assemblies on lipid vesicles reveal multiple orientations of the catalytic core with respect to the surface.

We described the formation of a complex between *PLCβ* and Gβγ as a two-step process: first, partitioning of *PLCβ* from aqueous solution into the membrane, and second, binding to Gβγ on the membrane surface. We explicitly consider two steps rather than one in which *PLCβ* binds directly to Gβγ for the following reasons. We measured partitioning of *PLCβ* into membranes without Gβγ and measured the corresponding catalysis of PIP2 in the absence of Gβγ . Thus, we know that *PLCβ* partitions onto the membrane surface without Gβγ . Furthermore, we find that *PLCβ* and Gβγ do not form a complex in the absence of a membrane, neither as evaluated by size exclusion chromatography (*SI Appendix*, Fig. S3*G*) nor on cryo-EM grids. It was also shown previously that Gβγ does not activate *PLCβ* in the absence of membranes (17). Taken together, this set of findings support the conclusion that *PLCβ* partitioning is a required first step in the two-step process of PLCβ3⋅Gβγ complex formation on membranes. We hypothesize that partitioning orients *PLCβ* with respect to Gβγ , defines a local surface concentration, and thus permits a binding equilibrium process that occurs in 2 dimensions, rather than in a three-dimensional aqueous phase.

We modeled the second step, the equilibrium reaction between PLCβ and Gβγ on the membrane surface, as bimolecular (1:1 stoichiometry) characterized by a single $K_{\mathrm{eq}}$ . In our structural analysis, however, we discovered two binding sites for Gβγ on PLCβ3 . Additional binding data, using multiple concentrations of Gβγ, for example, might reveal two distinct binding constants and whether they interact with each other (i.e., behave cooperatively). Such a finding would be important because multiple binding sites could shape the PLCβ3 activity response to GPCR stimulation. But for purposes of the present study, the binding model treating a single site is sufficient. This is because using a single site model when two sites exist introduces an uncertainty in how PLCβ3 is distributed over Gβγ , not how much PLCβ3 is present in the membrane. The kinetics depend on how much PLCβ3 is present, and this we have measured directly with experiment.

The conclusion that Gβγ concentrates PLCβ3 on the membrane in our assay is unequivocal. To what extent do these conclusions apply to cell membranes? From Eq. **8**, we saw that the increase in membrane PLCβ3 concentration due to the fraction bound to Gβγ is proportional to total Gβγ concentration, ${[G}_{\mathrm{tot}}]$ . In our assay, ${[G}_{\mathrm{tot}}]$ is 0.34 mol% , which corresponds to ~5,000 $G\beta \gamma {/\mu m}^{2}$ . In cells, we have previously estimated the concentration of Gβγ near GIRK2 channels in dopamine neurons during GABA_B_ receptor activation at ~1,200 $G\beta \gamma /{\mu m}^{2}$ (32). Applying Eq. **8**, this would produce an ~nine-fold increase in the membrane concentration of PLCβ3 . This is an estimate with certain unknowns, especially the cytoplasmic concentration of PLCβ3 ( $\left[{PLC\beta 3}_{w}\right]$ ), but the result suggests that the conditions of our in vitro assay are applicable to cell membranes. Moreover, both Gβγ and ${G\alpha }_{q}$ have been shown to increase membrane association of PLCβs in cells, consistent with our results (29).

We note that our demonstration that Gβγ increases membrane association of PLCβ3 directly contradicts many previous biochemical studies and the current consensus in the field that G proteins do not increase the local concentration of *PLCβs* in the membrane (3, 4, 22–24, 26–28). We suspect that the use of detergent solubilized Gβγ in past studies may have interfered with the control of its concentration on the membrane (22–24).

While our results and mechanism contradict the notion that G proteins do not concentrate PLCβs on the membrane, they are consistent with many previous observations, some we list here. As stated above, studies with cells have led to the conclusion that Gβγ and ${G\alpha }_{q}$ increase membrane association of PLCβs (29). The lipid anchor is required for the activation of *PLCβs* by the small GTPases and Gβγ , and G proteins do not activate *PLCβs* in the absence of a membrane environment (9–11, 13, 15, 17, 29). The binding of Rac1 or ${G\alpha }_{q}$ do not induce conformational changes around the active site, suggesting that activation is not mediated by obvious allosteric changes (9, 18, 19). Likewise, we observe no change in the PLCβ3 active site conformation when Gβγ is bound, only that Gβγ recruits PLCβ3 to the membrane and orients its active site.

Several properties of the Gβγ binding sites on PLCβ3 offer explanations of past observations. First, it has been shown that Gβγ and ${G\alpha }_{q}$ can activate PLCβ3 simultaneously (36, 37, 45–49). We find here that the Gβγ sites do not occlude the ${G\alpha }_{q}$ binding site (18, 19), and therefore both G proteins can in principle bind to PLCβ3 at the same time and activate PLCβ3 (3, 36, 37, 45). Second, several amino acids on Gβγ that contact *PLCβ*3 in the structure were previously shown to play a role in binding to Gα , *PLCβ*, and other effectors (Fig. 5 *C* and *D*) (43). Third, the PH domain was shown to play a role in Gβγ binding and activation; however, based on our structures, Gβγ binding does not require or induce rearrangement of the catalytic core as was previously proposed (50, 51). Fourth, Rac1 was also shown to bind to the PH domain of *PLCβ*2 (*SI Appendix*, Fig. S6*D*), and Rac1-activated *PLCβ* was shown to be additionally activated by Gβγ , leading to a proposal that the two binding sites did not overlap (9, 10). Our structures show that Rac1 and Gβγ do indeed share an interface within the PH domain (*SI Appendix*, Fig. S6*D*); however, the second Gβγ binding site can explain the dual activation (9, 10).

An intriguing aspect of PLCβ enzymes is that all wild-type structures show that the active site is occluded by the inhibitory X–Y linker. This includes complexes with ${G\alpha }_{q}$, Rac1 and, now, Gβγ (9, 16, 18, 19). It has been proposed that lipids are required to remove the X–Y linker to achieve catalysis (3, 16, 17). This must be true to some extent because unless the linker is displaced, even if only rarely, catalysis cannot occur. From our data, we put forth an alternative proposal that the active site is predominantly autoinhibited, accounting for a small $k_{\mathrm{cat}}$ , even in the presence of lipids. Consequently, in the absence of GPCR stimulation, the baseline partitioning of PLCβ enzyme from the cytoplasm to the membrane, determined by $K_{x}$ and the cytoplasmic concentration of PLCβ , will produce very little PIP2 hydrolysis. Only upon GPCR stimulation, when a large quantity of PLCβ partitions into the membrane, determined by $K_{\mathrm{eq}}$ and the Gβγ concentration generated by GPCR stimulation, is there enough PLCβ enzyme in the membrane, even though $k_{\mathrm{cat}}$ remains low, to catalyze PIP2 hydrolysis. In other words, a small $k_{\mathrm{cat}}$ combined with an ability to enact large changes in membrane enzyme concentration upon GPCR stimulation permits a strong signal when the system is stimulated and a minimal baseline when it is not.

## Materials and Methods

### Protein Expression, Purification, and Reconstitution.

All proteins were purified according to previously established protocols using affinity chromatography and size exclusion chromatography. Detailed methods are described in *SI Appendix*, *Materials and Methods: Protein Expression and Purification and Protein Reconstitution*.

### *PLCβ*3 Functional Assay.

*PLCβ* activity was measured using a planar lipid bilayer setup and a PIP2-dependent ion channel to report PIP2 concentration in the membrane over time. Detailed methods are described in *SI Appendix*, *Materials and Methods: Bilayer Experiments and Analysis*.

### Membrane Partitioning Experiments.

Fluorescently labeled *PLCβ*3 was mixed with LUVs and pelleted. Protein in the pellet and supernatant were quantified using fluorescence. Detailed methods are described in *SI Appendix*, *Materials and Methods: PLCβ3 Vesicle Partition Experiments*.

### *PLCβ*3 Structure Determination.

*PLCβ*3 was mixed with liposomes with or without *Gβγ* prior to sample vitrification. Cryo-EM data were collected using a Titan Krios with a Gatan K3 direct electron detector according to the parameters in *SI Appendix*, Table S1 and analyzed according to the procedures outlined in *SI Appendix*, Figs. S3–S5 and S7. Atomic models from previously determined structures were fit into our density maps, refined using PHENIX real-space refine (52), and manually adjusted. Detailed methods are described in *SI Appendix*, *Materials and Methods: Cryo-EM Sample Preparation and Data Collection, Cryo-EM Data Processing, and Model Building and Validation*.

## Supplementary Material

Appendix 01 (PDF)

## Data Availability

Cryo-EM maps and atomic models for all structures described in this work have been deposited to the Electron Microscopy Data Bank (EMDB) and the Protein Data Bank (PDB), respectively. Accession codes are as follows: *PLCβ*3 in solution-8EMV and EMD-28266, *PLCβ*3 in complex with *Gβγ* on vesicles-8EMW and EMD-28267, and *PLCβ*3 in complex with *Gβγ* on nanodiscs-8EMX and EMD-28268.
