# Supplementary material for: Gβγ activates PIP2 hydrolysis by recruiting and orienting PLCβ on the membrane surface
Source: Proc Natl Acad Sci U S A. 2023 May 12;120(20):e2301121120. doi: 10.1073/pnas.2301121120 (PMC10194004; doi:10.1073/pnas.2301121120)
Supplement: Supplementary file 1 — Appendix 01 (PDF) [file pnas.2301121120.sapp.pdf]

PNAS

Supporting Information for

$G\beta\gamma$ activates $PIP2$ hydrolysis by recruiting and orienting $PLC\beta$ on the membrane urface

Maria E. Falzone[a,b] and Roderick MacKinnon[a,b*]

[a]Laboratory of Molecular Neurobiology and Biophysics, The Rockefeller University, New York, NY, 10065. [b]Howard Hughes Medical Institute, The Rockefeller University, New York, United States, 10065.

*Correspondence to: Roderick MacKinnon

Email: mackinn@rockefeller.edu

This PDF file includes:

Figures S1 to S7
Tables S1 to S3
SI Materials and Methods
SI Appendix 2 (Mathematica notebook for derivation of Equation 7)
SI References

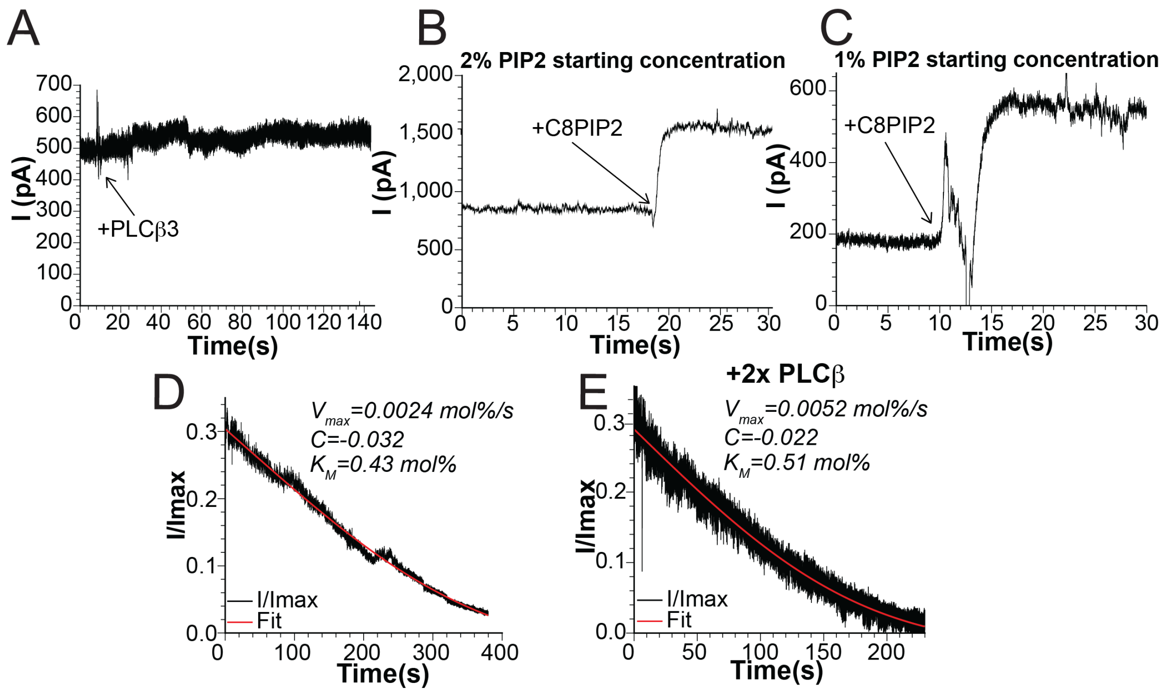

Figure S1. Planar lipid bilayer assay for $PLC\beta$ function. A: Representative current trace in the absence of $CaCl_2$ (2 mM EGTA) showing that addition of $PLC\beta3$ does not lead to current decay without Ca^{2+}, which is required for catalytic activity. B-C: Representative titration experiments for GIRK with $PIP2$ starting with 2 mol% (B) or 1 mol% (C). C-D: normalized current decay in the absence of $G\beta\gamma$ with 1x (D) or 2x (E) $PLC\beta3$ concentration fit to equation 4 showing that V_{max} depends on the added enzyme concentration. R^2=0.992 for D. R^2=0.969 for E.

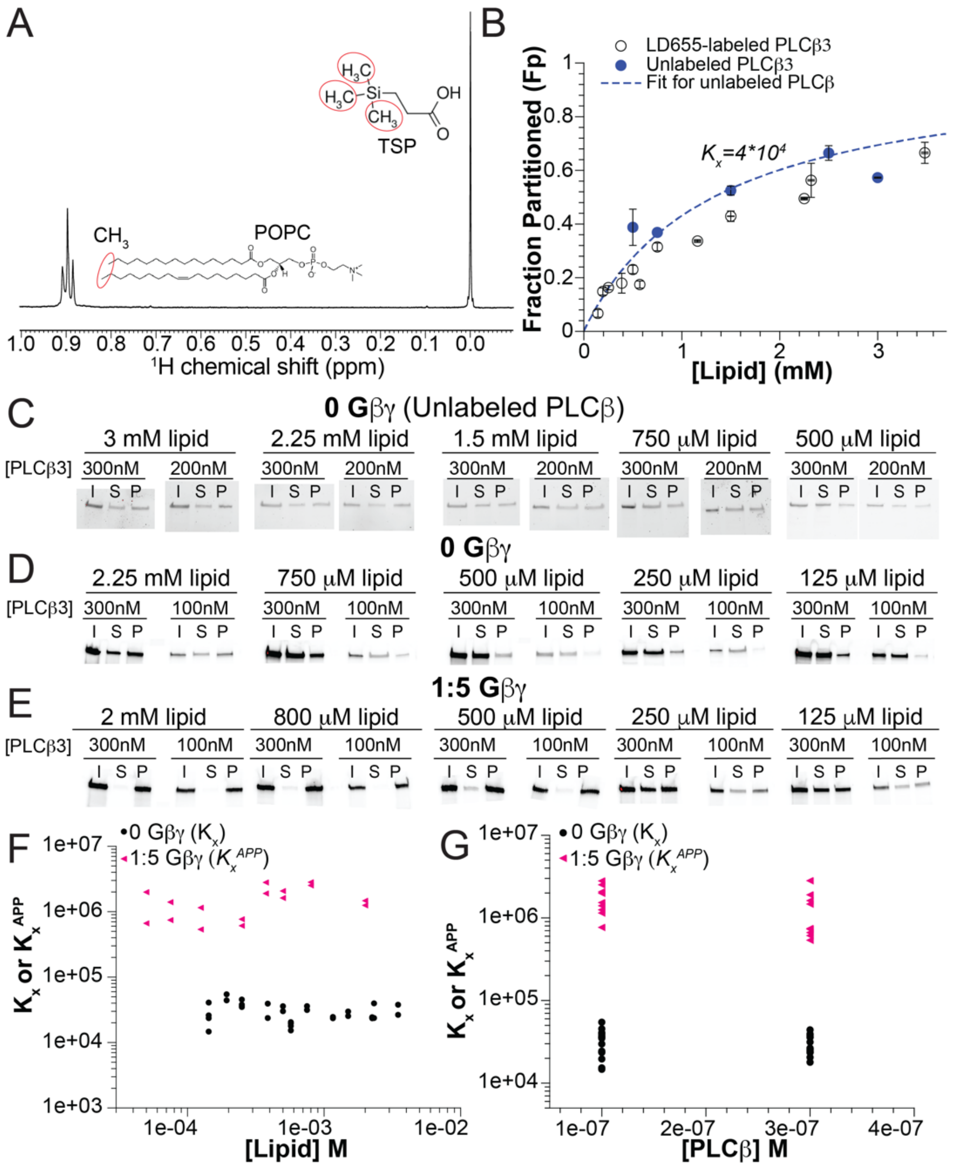

Figure S2. $G\beta\gamma$ enhances membrane partitioning of $PLC\beta3$. A: Representative ^{1}H NMR spectrum showing the lipid methyl peak at 0.9 ppm and the TSP peak at 0 ppm. Corresponding methyl groups are circled in red on the chemical structures. POPC is shown as a representative lipid. B: Membrane partitioning curve for $PLC\beta3$ without $G\beta\gamma$ labeled with LD655 (black) or unlabeled (blue) for 2DOPE:1POPC:1POPS LUVs. Data for labeled $PLC\beta3$ are reproduced from Figure 3D for display purposes. Data for unlabeled $PLC\beta3$ were fit to Equation 6 for K_x (dashed blue line) (1). Error bars are range of mean from two experiments for each lipid concentration. R^2 =0.56. C-E: Example SDS-PAGE gels imaged using Bio-Rad Stain-Free protocol (C) or for LD655 fluorescence (D-E) from binding experiments with (E) or without $G\beta\gamma$ (C-D). I represents input, S represents supernatant, and P represents pellet. F-G: Plot of individual partition coefficients in the absence of Gβγ (K_x) or apparent partition coefficients in the presence of $G\beta\gamma$ (K_x^{APP}) determined for each experiment plotted against lipid concentration (F) or against $PLC\beta3$ concentration (G) showing that values do not vary with concentration of lipid (F) or $PLC\beta3$ (G). Experiments without $G\beta\gamma$ are shown as black spheres and experiments with $G\beta\gamma$ are shown as pink triangles.

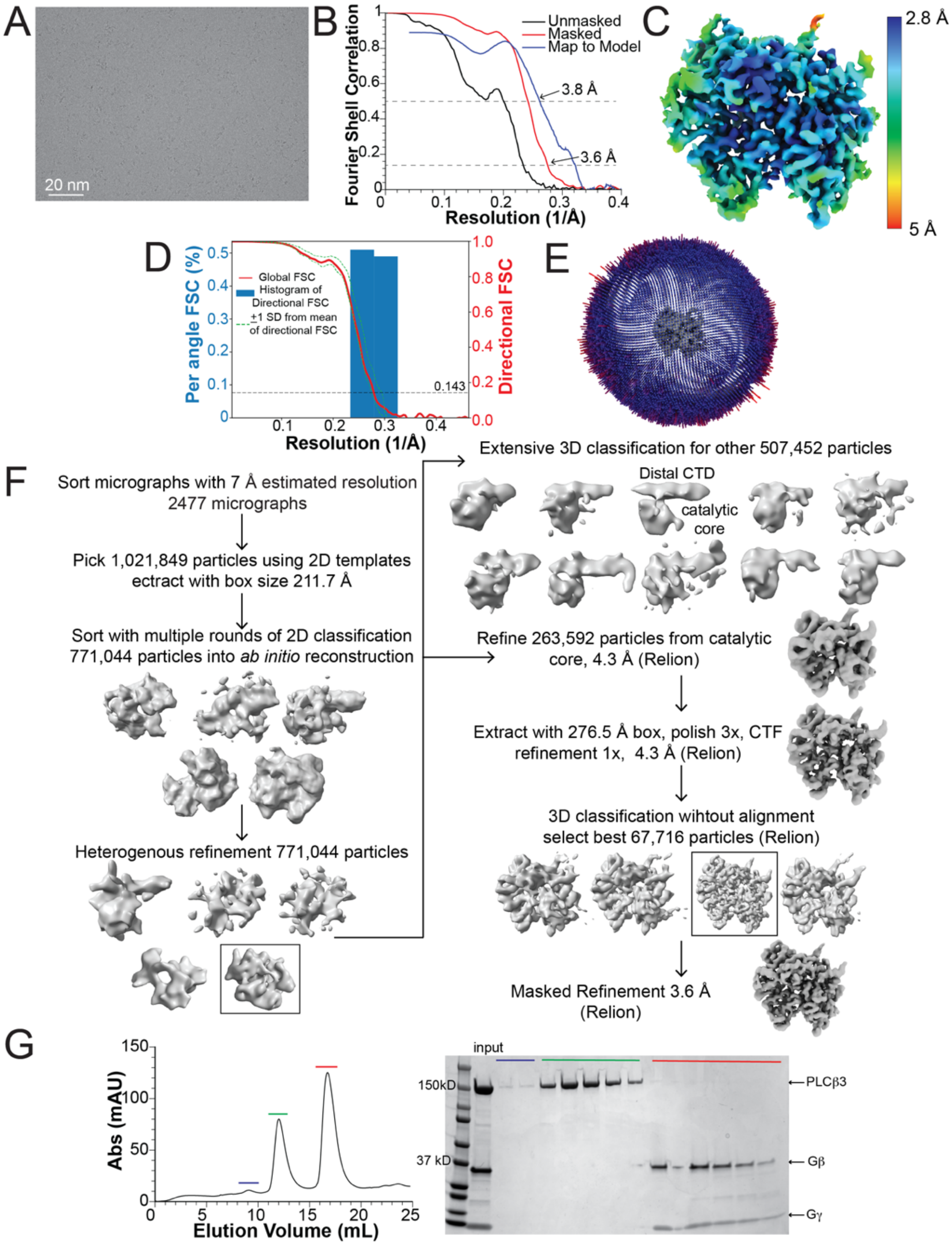

Figure S3. Structure determination of $PLC\beta3$ in solution without membranes. A: Representative micrograph. B: Fourier shell correlation (FSC) curves for the unmasked (black) and masked (red) maps and between the map and model (blue). The 0.143 and 0.5 thresholds are denoted by dashed lines. C: Final masked, sharpened map colored by local resolution determined by cryoSPARC. D: 3D FSC plot for the final masked map (2). E: Angular distribution plot for the final masked map from RELION. F: Summary of data processing steps, see methods. Maps shown are unsharpened. G: Attempt to form complex between $PLC\beta3$ and soluble $G\beta\gamma$ C68S. Proteins were mixed at a 1:2 ratio ($PLC\beta3$:$G\beta\gamma$) and ran on gel filtration using a Superdex 200 increase column (left). Peaks are color coded to SDS-PAGE gel (right) showing $PLC\beta3$ and $G\beta\gamma$ do not comigrate.

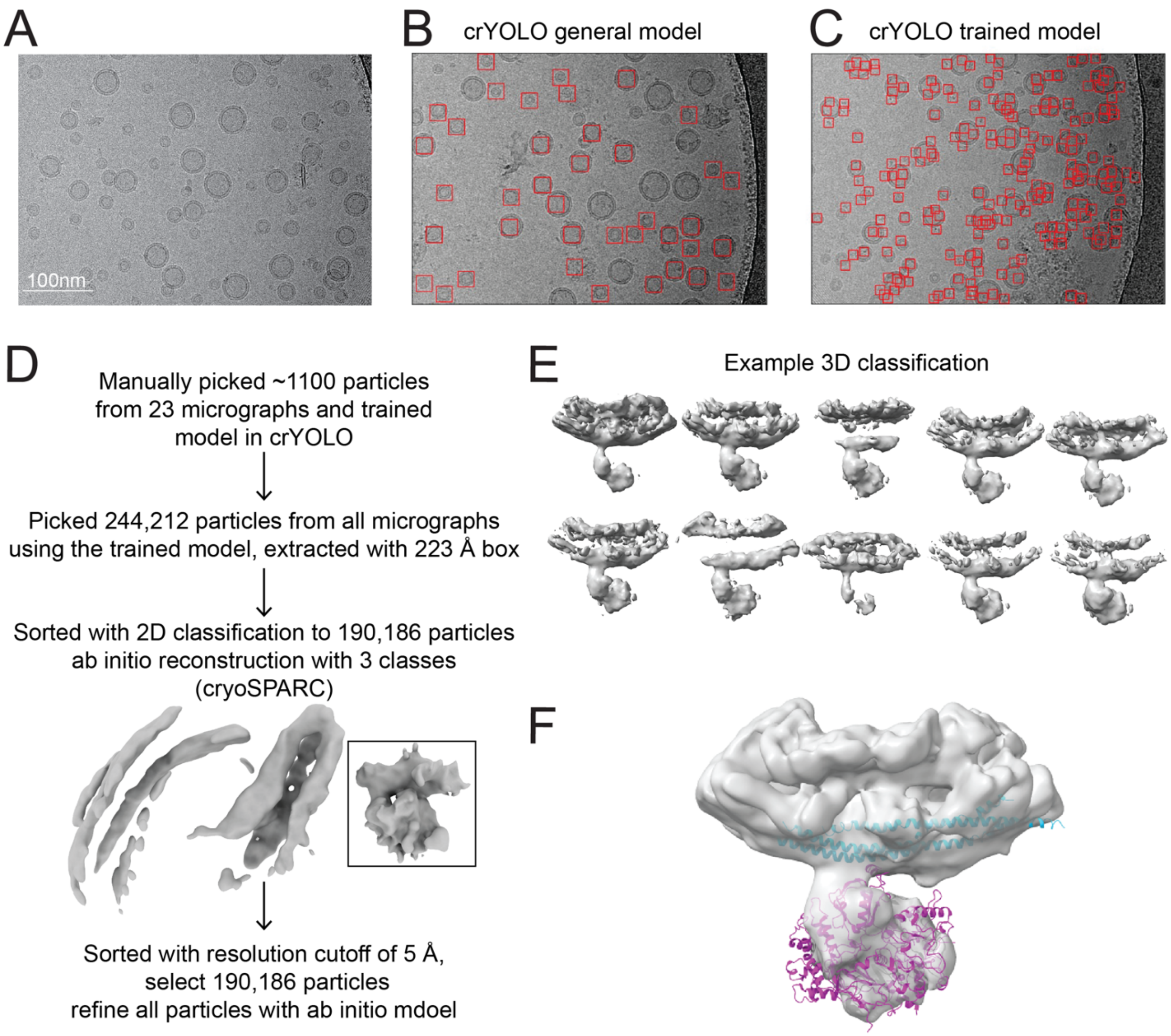

Figure S4. Structure studies of $PLC\beta3$ associated with liposomes. A: Representative micrograph. B-C: Example of particle picking using the crYOLO general model (C) or a crYOLO model trained on these micrographs (D) (3). Picked particles are shown as a red box. D: Summary of data processing steps, see methods. E: Example of 3D classification of the final model run in cryoSPARC without alignment. F: Final unsharpened map with models for the distal CTD (blue, PDBID 4GNK (4)) and the catalytic core (pink, PDBID 4GNK (4)) fit into the density. Extra density at the top is the membrane.

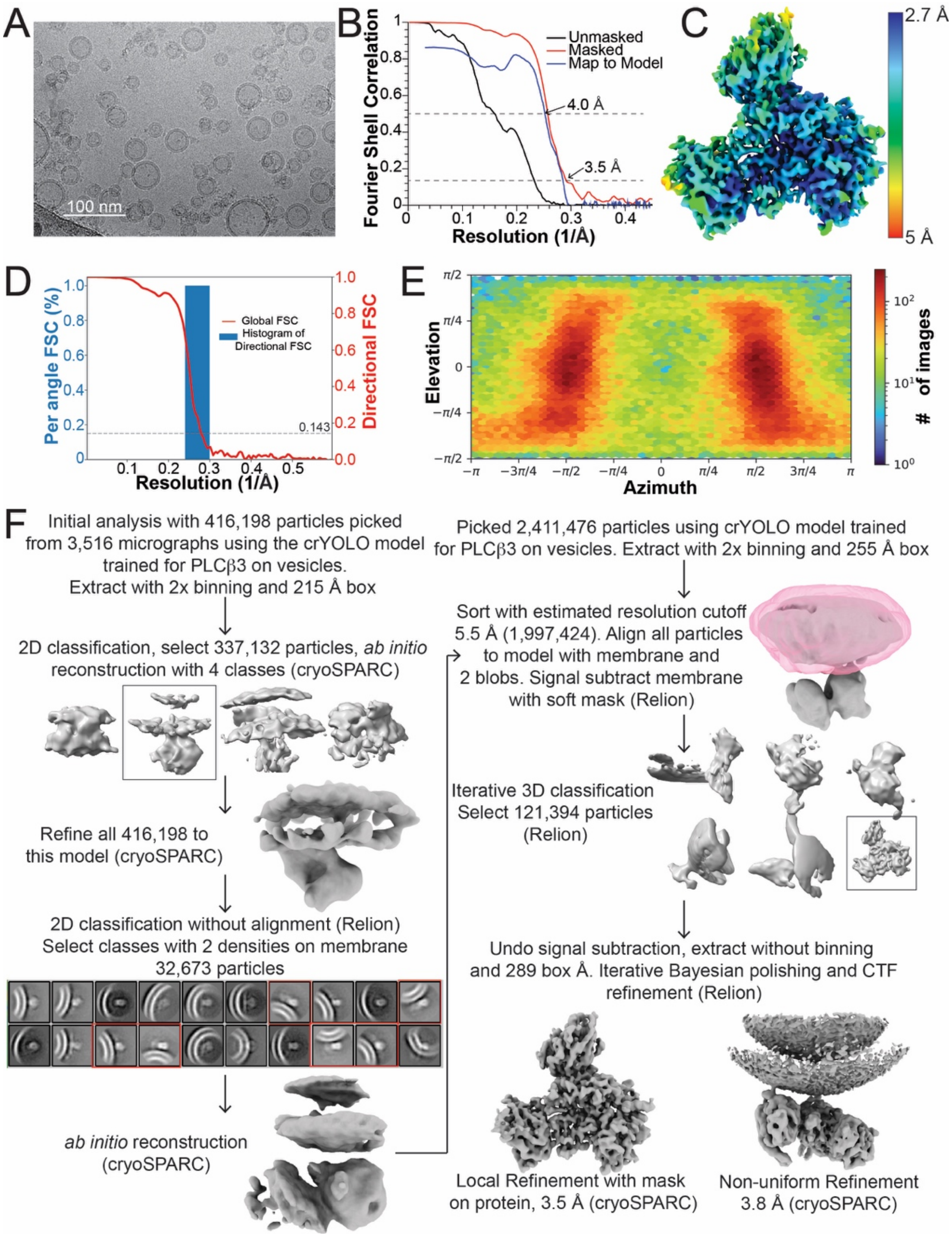

Figure S5. Structure determination of the $PLC\beta3 \cdot G\beta\gamma$ complex on liposomes comprised of 2DOPE:1POPC:1POPS. A: Representative micrograph. B: Fourier shell correlation (FSC) curves for the unmasked (black) and masked (red) maps and between the map and model (blue). The 0.143 and 0.5 thresholds are denoted by dashed lines. C: Final masked, sharpened map colored by local resolution determined by cryoSPARC. D: 3D FSC plot for the final masked map (2). E: Angular distribution plot for the final masked map from cryoSPARC. F: Summary of data processing steps, see methods. Maps shown are unsharpened.

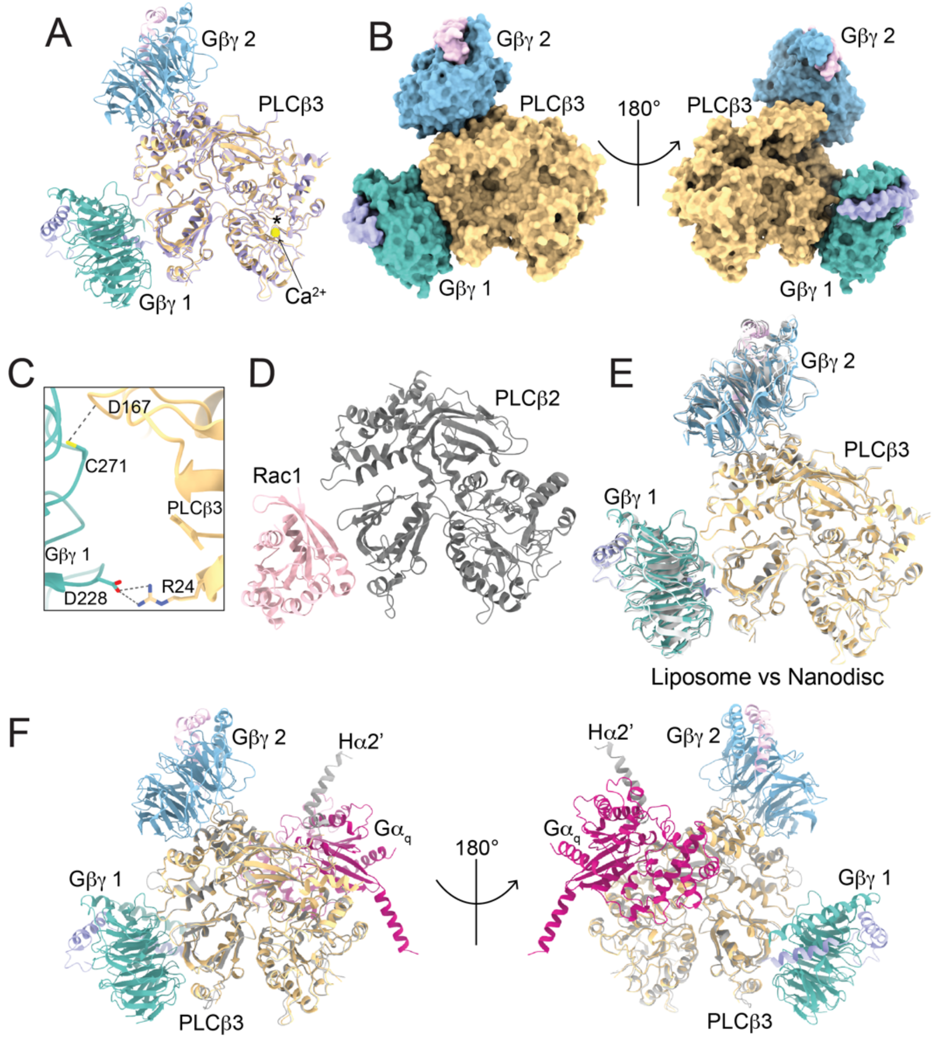

Figure S6. Interfaces of the $PLC\beta3 \cdot G\beta\gamma$ complex. A: Structural alignment of the $PLC\beta3$ catalytic core determined by cryo-EM (purple) and the $PLC\beta3 \cdot G\beta\gamma$ complex (colored by protein). rmsd ~0.7 Å. $PLC\beta3$ is yellow, $G\beta1$ is dark teal, $G\gamma1$ is light purple, $G\beta2$ is light blue and $G\gamma2$ is light pink. Calcium ion is shown as a yellow sphere and active site is denoted by asterisk. B: Surface representation of the $PLC\beta3 \cdot G\beta\gamma$ complex viewed from the top (left) and bottom (right) highlighting the $PLC\beta3$-$G\beta\gamma$ interfaces, colored as in A. C: Hydrogen bonds in the $G\beta\gamma1$ interface, $PLC\beta3$ is yellow and $G\beta$ is dark teal. D: $PLC\beta2$ catalytic core (gray) in complex with Rac1 (pink) highlighting similar positioning of $G\beta\gamma1$. PDBID-2FJU (5). E: Structural alignment of the $PLC\beta3 \cdot G\beta\gamma$ complex on liposomes (colored by protein) and on nanodiscs (gray). rmsd ~0.8 Å. F: Structural alignment of the $PLC\beta \cdot 3G\beta\gamma$ complex to the crystal structure of the $PLC\beta3 \cdot Ga_q$ complex PDBID-4QNK (4). The $PLC\beta \cdot 3G\beta\gamma$ complex is colored as in panel A and in the $PLC\beta3 \cdot Ga_q$ complex, the PLCβ catalytic core is gray and $G\alpha_q$ is pink.

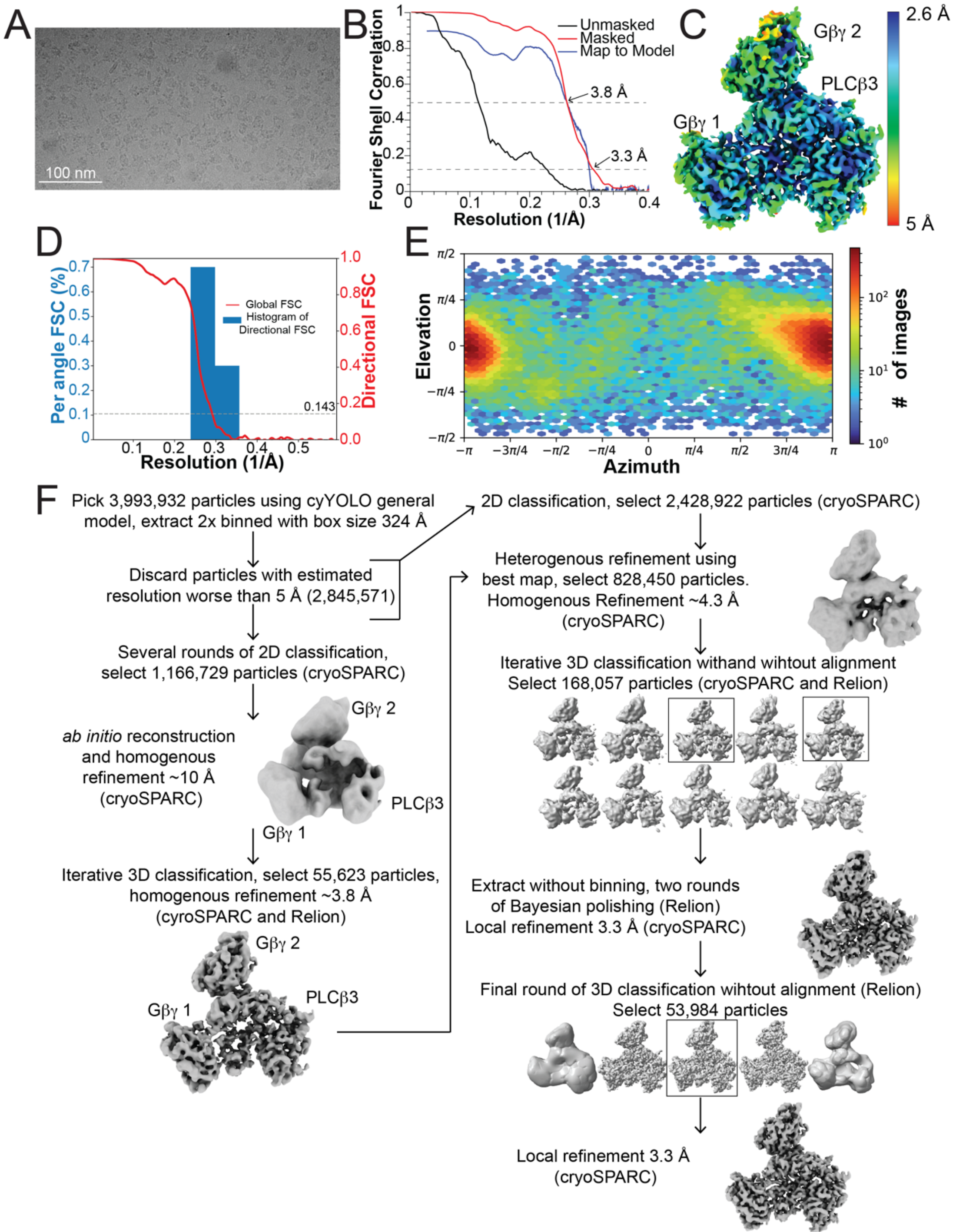

Figure S7. Structure determination of $PLC\beta3 \cdot G\beta\gamma$ complex on nanodiscs A: Representative micrograph. B: Fourier shell correlation (FSC) curves for the unmasked (black) and masked (red) maps and between the map and model (blue). The 0.143 and 0.5 thresholds are denoted by dashed lines. C: Final masked, sharpened map colored by local resolution determined by cryoSPARC. D: 3D FSC plot for the final masked map (2). E: Angular distribution plot for the final masked map from cryoSPARC. F: Summary of data processing steps, see methods. Maps shown are unsharpened.

Table S1. Cryo-EM collection parameters and model statistics.

Collection Parameters	$PLC\beta3$ solution	$PLC\beta3/G\beta\gamma$ complex-liposomes	$PLC\beta3/G\beta\gamma$ complex-nanodiscs	$PLC\beta3$ liposomes
Accelerating Voltage (kV)	300	300	300	300
Number of frames	40	50	40	50
Dose ($e^-/Å^2$)	42.87	60	69.14	50.7
Defocus Range (μm)	1-2.5	1.5-2.5	1-2.5	1.5-2.5
Exposure Time (s)	2	4.194	2	1.5
Original Pixel size (Å)	0.54	0.4195	0.844	0.435
Map Parameters				
Final Pixel Size (Å)	1.08	0.839	0.844	0.87
Symmetry	C1	C1	C1	C1
Total micrographs	3,527	27,454	25,063	5,448
Initial particles	1,021,849	1,997,424	1,728,771	244,212
Final particles	67,716	121,394	53,984	244,212
Map resolution (Å)	3.59	3.47	3.33	
FSC threshold	0.143	0.143	0.143	
Map local resolution range (Å)	2.8-5.0	2.7-5.0	2.6-5.0	
Map sharpening B factor ($Å^2$)	-111.735	-116	-79.1	
Initial Model Used	4GNK	8EMV	8EMV	
Model resolution (Å) (FSC_{model}=0.5)	3.8	4.0	3.8	
Model Composition				
Nonhydrogen atoms	5,949	11,732	11,735	
Protein residues	747	1506	1506	
Ligands	1	1	1	
r.m.s. deviations bond length (Å)	0.007	0.008	0.006	
r.m.s. deviations bond length (Å)	0.8	0.899	0.798	
Validation				
MolProbity Score	1.53	1.77	1.59	
Clash Score	3.11	6.63	4.73	
Poor Rotamers (%)	0	0	0	
Ramachandran Plot				
Favored (%)	93.50	93.88	94.95	
Allowed (%)	6.5	6.12	5.05	
Disallowed (%)	0	0	0	

Table S2. Interface residues in the $PLC\beta3/G\beta\gamma$ complex. Residues identified have buried surface area > 15 Å^2. W99, M101, L117, T143, D186, D228, W332 from $G\beta$ were shown to be important for $PLC\beta$ activation (6).

Interface	Protein	Residues
$G\beta\gamma 1$	$PLC\beta3$	R24, K27, I29, R38, N39, L40, P57, N58, M59, V89, R204, V123, Q166, D167, G168, and R169
	$G\beta 1$	K57, S98, W99, M101, L117, Y145, C204, D228, D267, N267, I270, C271, D290, D291, F292, N313, R314, and W332
$G\beta\gamma 2$	$PLC\beta3$	R185, T188, S192, R215, N218, K219, L222, P224, D227, L231, K236, G237, K238, P239, Y240, N282, Q284, F285, R288, M293, and E294
	$G\beta 2$	L55, A56, K57, Q75, D76, K78, S98, W99, M101, L117, T143, Y145, D186, M188, C204, D228, N230, D246, D290, N313, R314, W332, and D333

Table S3: Information for critical reagents

Reagent	Source	Catalog number
Sodium cholate hydrate	Sigma	Cat# C6445
Bio-Beads SM-2 Resin	Bio-rad	Cat# 1523920
n-Decyl-β-D-Maltopyranoside	anatrace	Cat# D322S
n-Dodecyl-β-D-Maltopyranoside	anatrace	Cat# D310S
Fos-Choline-8, Fluorinated	anatrace	Cat# F300F
LD655-maleimide	Lumidyne technologies	Cat# 09
1,2-dioctanoyl-sn-glycero-3-phospho-(1'-myo-inositol-4',5'-bisphosphate) (C8-PI(4,5)P_2)	Avanti polar lipids	Cat# 850185
1-palmitoyl-2-oleoyl-sn-glycero-3-phosphoethanolamine (POPE)	Avanti polar lipids	Cat# 850757C
1-palmitoyl-2-oleoyl-sn-glycero-3-phospho-(1'-rac-glycerol) (sodium salt) (POPG)	Avanti polar lipids	*Cat# 840457C*
1,2-dioleoyl-sn-glycero-3-phosphoethanolamine (DOPE)	Avanti polar lipids	Cat# 850725C
1-palmitoyl-2-oleoyl-glycero-3-phosphocholine (POPC)	Avanti polar lipids	Cat# 850457C
1-palmitoyl-2-oleoyl-sn-glycero-3-phospho-L-serine (sodium salt) (POPS)	Avanti polar lipids	Cat# 840034C
1,2-dioleoyl-sn-glycero-3-phosphoethanolamine-N-(lissamine rhodamine B sulfonyl) (ammonium salt) (18:1 Liss Rhod PE)	Avanti polar lipids	Cat# 810150C
L-α-phosphatidylinositol-4,5-bisphosphate (Brain, Porcine) (ammonium salt) (Brain PI(4,5)P2)	Avanti polar lipids	Cat# 840046X
Penicillin-Streptomycin (10,000 U/mL)	Thermo Fisher	Cat# 15140122
Cellfectin II Reagent	Thermo Fisher	Cat# 10362100
Fetal bovine serum	Thermo Fisher	Cat# 16000044
Penicillin-Streptomycin (10,000 U/mL)	Thermo Fisher	Cat# 15140122
L-Glutamine (200 mM)	Thermo Fisher	Cat# 25030081
FreeStyle 293 expression medium	Thermo Fisher	Cat# 12338018
Express Five SFM	Thermo Fisher	Cat# 10486025
Sf-900 II SFM	Thermo Fisher	Cat# 10902096
CNBR-activated Sepharose 4B	Cytiva	Cat# 17043002
Superose 6 Increase 10/300 GL	Cytiva	Cat# 29091569
Superdex 200 Increase 10/300 GL	Cytiva	Cat# 28990944
KLD enzyme mix	NEB	Cat# M0554S
NEBuilder® HiFi DNA Assembly Master Mix	NEB	Cat# E2621S

SI Materials and Methods

Preparation of GFP nanobody-coupled Sepharose resin

The enhancer nanobody (7) with a c-terminal hexa-histidine tag in a pET32a plasmid was transformed into BL21 (DE3) competent cells. After recovery, the transformed cells were added directly to LB with 100 $\mu g/mL$ ampicillin and grown overnight. The starter culture was used to inoculate large cultures (also with 100 $\mu g/mL$ ampicillin), which were grown to an OD600 of 0.9 at 37°C. Expression was induced with 0.5 mM isopropyl-β-D-thiogalactoside (IPTG) and the temperature was reduced to 20°C for ~18 hours. Cells were harvested by centrifugation at 3,500 x g for 15 minutes. Pellets were flash frozen and stored at -80°C until use. Purification was carried out at 4°C. Cells were resuspended in 50 mM Na-phosphate pH 7.5, 300 mM NaCl, 10% glycerol (v/v) supplemented with DNase and protease inhibitors (12.5 $\mu g/mL$ leupeptin, 12.5 $\mu g/mL$ pepstatin A, 625 $\mu g/mL$ AEBSF, 1 mM Benzamadine, 100 $\mu g/mL$ Trypisin inhibitor, 1x aprotinin, and 1 mM PMSF) and lysed by brief sonication. Lysate was clarified by centrifugation at 39,000 x g for 45 minutes, supplemented with 5 mM Imidazole, and bound in batch to 10 mL TALON resin equilibrated with buffer containing 50 mM Na-phosphate pH 7.5, 300 mM NaCl, 10% glycerol (v/v), and 5 mM imidazole. The resin was washed in batch with 100 mL of equilibration buffer then loaded onto a column and washed by gravity flow with 10 column volumes of 10 mM imidazole buffer (50 mM Na-phosphate pH 7.5, 300 mM NaCl, 10% glycerol (v/v) and 10 mM imidazole). Protein was eluted with ~50 mL buffer containing 50 mM Na-phosphate pH 7.5, 300 mM NaCl, 10% glycerol and 200 mM imidazole and the first 8 mL of elution were discarded. The remaining protein was concentrated to ~10 mL using a 15-mL Amicon concentrator with 10-kDa molecular weight cutoff and dialyzed overnight in 50 mM Na-phosphate pH 7.5, 300 mM NaCl.

Protein was coupled to CNBr-Activated Sepharose 4 Fast Flow resin according to the manufacturer's protocol. 100 mg of protein was coupled to 25 g of resin which resulted in a final 200 mL of 50% slurry. Resin was prepared by mixing with 1 mM cold HCl, manually breaking up chunks, loaded onto a column and washed with additional cold HCl. The resin was then washed with cold coupling buffer (100 mM $NaHCO_3$, and 500 mM NaCl) and transferred to a bottle. The nanobody was diluted into coupling buffer, added to the resin, and rotated overnight at 4°C. The resin was pelleted by spinning for 10 minutes at 2,000 g and washed with additional coupling buffer. The discarded supernatant was checked for protein and the resin was spun again, resuspended in blocking buffer (50 mM NaPhosphate pH 8.0, 150 mM NaCl, 50 mM glycine), and rotated at room temperature for 2 hours. The resin was washed extensively with wash buffer (10 mM NaPhosphate pH 7.0 and 150 mM NaCl) and finally stored at 4°C as a 50% slurry in wash buffer.

Protein Expression and Purification

The human $PLC\beta3$ gene was provided by Dr. Sondek (8) and contained residues 10 to 1234. It was cloned into the pFastBac vector downstream of GFP and transformed into DH10bac cells to produce bacmid DNA, which was used to transfect Sf9 cells to produce

baculovirus. High Five insect cells at 2x10^6 cells/mL were infected with 15-25 mL of P3 virus per liter of culture and harvested 36-48 hours after infection by centrifugation at 3,500 x g for 15 minutes. Pellets were flash frozen and stored at -80°C until use. Purification was carried out at 4°C. Cells were resuspended in 100 mL of buffer containing 50 mM HEPES pH 8.0, 50 mM NaCl, 10 mM 2-mercaptoethanol, 5% glycerol (v/v), 0.1 mM EDTA, 0.1 mM EGTA, DNase and protease inhibitors (12.5 $\mu g/mL$ leupeptin, 12.5 $\mu g/mL$ pepstatin A, 625 $\mu g/mL$ AEBSF, 1 mM Benzamadine, 100 $\mu g/mL$ Trypisin inhibitor, 1x aprotinin, and 1 mM PMSF) and lysed by brief sonication. Lysate was clarified by centrifugation at 39,000 x g for 45 minutes and bound to GFP nanobody-coupled Sepharose resin (prepared in-house) for one hour. The resin was washed in batch once with 10 column volumes of buffer containing 20 mM HEPES pH 8.0, 400 mM NaCl, 10 mM 2-mercaptoethanol, 2% glycerol (v/v), 0.1 mM EDTA, 0.1 mM EGTA, and protease inhibitors (625 $\mu g/mL$ AEBSF, 1 mM Benzamadine, 100 $\mu g/mL$ Trypisin inhibitor, and 1x aprotinin) then loaded onto a column and washed with an additional 10 column volumes by gravity flow. Protein was eluted by cleavage with 3C PreScission protease (made in house) for two hours, concentrated to ~10 mg/mL using a 15-mL Amicon concentrator with 100-kDa molecular weight cutoff, and further purified by size exclusion chromatography using a Superdex 200 10/300 increase column in buffer containing 20 mM HEPES pH 8.0, 100 mM NaCl, 5 mM Dithiothreitol (DTT), 2% glycerol (v/v), 0.1 mM EDTA, 0.1 mM EGTA, and protease inhibitors (12.5μg/mL leupeptin, 12.5μg/mL pepstatin A, 625μg/mL AEBSF, 1 mM Benzamadine, 100 μg/mL Trypisin inhibitor, 1x aprotinin, and 1 mM PMSF). Fractions with $PLC\beta$3 were pooled, flash frozen, and stored at -80°C for later use. For preparation of cryo-EM grids, protein was used directly after size exclusion.

To purify protein for non-specific cysteine labeling, the 10 mM 2-mercaptoethanol in all buffers was replaced with 2 mM tris(2-carboxyethyl)phosphine (TCEP) and the protein-loaded resin was washed with buffer containing 20 mM HEPES pH 7.4, 400 mM NaCl, 2 mM TCEP, 2% glycerol (v/v), 0.1 mM EDTA, 0.1 mM EGTA, and protease inhibitors (625 $\mu g/mL$ AEBSF, 1 mM Benzamadine). Following elution, maleimide LD655 (9) was added in 5-fold molar excess and incubated overnight protected from light. Labeled protein was concentrated ~10 mg/mL using a 15-mL Amicon concentrator with 100-kDa molecular weight cutoff, and further purified by size exclusion chromatography using a superdex 200 10/300 increase column in buffer containing 20 mM HEPES pH 8.0, 100 mM NaCl, 5 mM Dithiothreitol (DTT), 2% glycerol (v/v), 0.1 mM EDTA, 0.1 mM EGTA, and protease inhibitors 12.5 $\mu g/mL$ leupeptin, 12.5 $\mu g/mL$ pepstatin A, 625 $\mu g/mL$ AEBSF, 1 mM Benzamadine, 100 $\mu g/mL$ Trypisin inhibitor, and 1x aprotinin). Fractions with labeled $PLC\beta$3 were pooled, flash frozen, and stored at -80°C for later use. Labeling efficiency was consistently 60-70%.

For lipidated $G\beta\gamma$, untagged human $G\beta$1 was co-expressed with human $G\gamma$2 with an N-terminal His-YFP tag in High Five insect cells using 12 and 8 mL of P3 baculovirus respectively at 2x10^6 cells/mL for 36-48 hours. Cells were harvested by centrifugation at 3,500 x g for 15 minutes and pellets were flash frozen and stored at -80°C until use. Purification was carried out at 4°C. ~45 mL of cells were resuspended in 200 mL of buffer containing 25 mM Tris-HCl pH 8.0, 125 mM NaCl, 5 mM EGTA, 5 mM DTT, DNase and protease inhibitors (12.5 $\mu g/mL$ leupeptin, 12.5 $\mu g/mL$ pepstatin A, 625

$\mu g/mL$ AEBSF, 1 mM Benzamadine, 100 $\mu g/mL$ Trypisin inhibitor, 1x aprotinin, and 1 mM PMSF) and broken by manual homogenization. Membranes were separated by centrifuging at 39,000 x g for 30 minutes, resuspended in buffer containing 25 mM Tris-HCl pH 8.0, 125 mM NaCl, DNase and protease inhibitors (12.5 $\mu g/mL$ leupeptin, 12.5 $\mu g/mL$ pepstatin A, 625 $\mu g/mL$ AEBSF, 1 mM Benzamadine, 100 $\mu g/mL$ Trypisin inhibitor, 1x aprotinin, and 1 mM PMSF) and manually homogenized again. Proteins were extracted using 1% sodium cholate added from a 10% stock by mixing for 1.5 hours. Lysate was clarified by centrifuging at 39,000 x g for 30 minutes and bound in batch to 10 mL TALON resin equilibrated with buffer containing 25 mM Tris-HCl pH 8.0, 125 mM NaCl, and 1% sodium cholate for one hour. The resin was washed in batch with 100 mL of equilibration buffer then loaded onto a column and washed by gravity flow with 10 column volumes of high salt buffer (25 mM Tris-HCl pH 8.0, 500 mM NaCl, and 1% sodium cholate) and 10 mM imidazole buffer (25 mM Tris-HCl pH 8.0, 125 mM NaCl, 1% sodium cholate, and 10 mM imidazole). Protein was eluted with ~20 mL buffer containing 25 mM Tris-HCl pH 8.0, 125 mM NaCl, 1% sodium cholate, and 200 mM imidazole and concentrated to ~2 mL using a 15-mL Amicon concentrator with 30-kDa molecular weight cutoff. The final 2 mL of protein was diluted to ~20 mL using buffer without imidazole and incubated with 3C PreScission protease (prepared in-house) overnight at 4°C remove the His-YPF. The cleaved protein was passed over a column of 10 mL TALON resin equilibrated with 25 mM Tris-HCl pH 8.0, 125 mM NaCl, 1% sodium cholate, and 20 mM imidazole three times to remove the free His-YFP, and concentrated to 1 mL using a 15-mL Amicon concentrator with 30-kDa molecular weight cutoff. $G\beta\gamma$ was purified further via size exclusion chromatography using a Superdex 200 10/300 increase column in buffer containing 25 mM Tris-HCl pH 8.0, 125 mM NaCl, 1% sodium cholate, and 5 mM DTT. Fractions containing $G\beta\gamma$ were pooled and concentrated to 5-10 mg/mL using 4-mL Amicon concentrator with 30-kDa molecular weight cutoff and immediately used for reconstitution.

For nanodisc reconstitution, the YFP was maintained to facilitate purification of protein-containing nanodiscs. Following elution from TALON resin, protein was concentrated to 1 mL using a 15-mL Amicon concentrator with 30-kDa molecular weight cutoff and further purified by size exclusion chromatography using a Superdex 200 10/300 increase column in buffer containing 25 mM Tris-HCl pH 8.0, 125 mM NaCl, 1% sodium cholate, and 5 mM DTT. Fractions containing $G\beta\gamma-$YFP were pooled and concentrated to ~10 mg/mL using 4-mL Amicon concentrator with 30-kDa molecular weight cutoff and immediately used for reconstitution.

For soluble $G\beta\gamma$, untagged human $G\beta$1 was co-expressed with human $G\gamma$2 C68S with an N-terminal His-YFP tag in High Five insect cells using 12 and 8 mL of P3 baculovirus respectively at $2x10^6$ cells/mL for 36-48 hours. Cells were harvested by centrifugation at 3,500 x g for 15 minutes and pellets were flash frozen and stored at -80°C until use. Purification was carried out at 4°C. ~45 mL of cells were resuspended in 200 mL of buffer containing 25 mM Tris-HCl pH 8.0, 125 mM NaCl, DNase and protease inhibitors (12.5 $\mu g/mL$ leupeptin, 12.5 $\mu g/mL$ pepstatin A, 625 $\mu g/mL$ AEBSF, 1 mM Benzamadine, 100 $\mu g/mL$ Trypisin inhibitor, 1x aprotinin, and 1 mM PMSF) and broken brief sonication. Lysate was clarified by centrifuging at 39,000 x g for 45 minutes and bound in batch to 10 mL TALON resin equilibrated with buffer containing 25 mM Tris-HCl pH 8.0, and 125 mM NaCl for one hour. The resin was

washed in batch with 100 mL of equilibration buffer then loaded onto a column and washed by gravity flow with 10 CV of high salt buffer (25 mM Tris-HCl pH 8.0, and 500 mM NaCl) and 10 mM imidazole buffer (25 mM Tris-HCl pH 8.0, 125 mM NaCl, and 10 mM imidazole). Protein was eluted with ~20 mL buffer containing 25 mM Tris-HCl pH 8.0, 125 mM NaCl, and 200 mM imidazole and concentrated to ~2 mL using a 15-mL Amicon concentrator with 30-kDa molecular weight cut off. The final 2 mL of protein was diluted to ~20 mL using buffer without imidazole and incubated with 3C PreScission protease (prepared in-house) overnight at 4°C to remove the His-YPF. The cleaved protein was passed over a column of 10 mL TALON resin equilibrated with 25 mM Tris-HCl pH 8.0, 125 mM NaCl, and 20 mM Imidazole three times to remove the free His-YFP, and concentrated to 1 mL using a 15-mL Amicon concentrator with 30-kDa molecular weight cutoff. $G\beta\gamma$ was purified further using size exclusion chromatography using a superdex 200 10/300 increase column in buffer containing 25 mM Tris-HCl pH 8.0, 125 mM NaCl, and 5 mM DTT. Fractions containing $G\beta\gamma$ were pooled, flash frozen, and stored at -80°C until use.

To attempt to form a complex between $PLC\beta3$ and soluble $G\beta\gamma$, proteins were mixed at a 2:1 molar ratio of $G\beta\gamma : PLC\beta3$, incubated on ice for one hour and run on size exclusion chromatography using a Superdex 200 10/300 increase column in buffer containing 20 mM HEPES pH 8.0, 100 mM NaCl, 5 mM Dithiothreitol (DTT), 2% glycerol (v/v), 0.1 mM EDTA, 0.1 mM EGTA, and protease inhibitors (112.5 $\mu g/mL$ leupeptin, 12.5 $\mu g/mL$ pepstatin A, 625 $\mu g/mL$ AEBSF, 1 mM Benzamadine, 100 $\mu g/mL$ Trypisin inhibitor, 1x aprotinin, and 1 mM PMSF). Peaks were analyzed by SDS-PAGE and showed no-comigration (Figure S3G).

For ALFA-nanobody tagged $G\beta\gamma$, untagged human $G\beta1$ was co-expressed with human $G\gamma2$ with the ALFA nanobody on the C-terminus and an N-terminal His-YFP tag in High Five insect cells using 12 and 8 mL of P3 baculovirus respectively at 2x10^6 cells/mL for 36-48 hours. Cells were harvested by centrifugation at 3,500 x g for 15 minutes and pellets were flash frozen and stored at -80°C until use. Purification was carried out at 4°C. ~45 mL of cells were resuspended in 200 mL of buffer containing 25 mM Tris-HCl pH 8.0, 125 mM NaCl, 5 mM DTT, DNase and protease inhibitors (12.5 $\mu g/mL$ leupeptin, 12.5 $\mu g/mL$ pepstatin A, 625 $\mu g/mL$ AEBSF, 1 mM Benzamadine, 100 $\mu g/mL$ Trypisin inhibitor, 1x aprotinin, and 1 mM PMSF) and broken by brief sonication. Lysate was clarified by centrifugation at 39,000 x g for 45 minutes and bound to GFP nanobody-coupled Sepharose resin (prepared in house) for one hour. The resin was washed in batch one time with 10 column volumes of buffer containing 25 mM Tris-HCl pH 8.0, 125 mM and NaCl, then loaded into a column and washed with an additional 10 column volumes by gravity flow. Protein was eluted by cleavage with 3C PreScission protease (prepared in-house) for two hours, concentrated to 1 mL using a 15-mL Amicon concentrator with 30-kDa molecular weight cutoff, and further purified by size exclusion chromatography using a Superdex 200 10/300 increase column in buffer containing 25 mM Tris-HCl pH 8.0, 125 mM NaCl, and 5 mM DTT. Fractions with nanobody-tagged $G\beta\gamma$ were pooled, flash frozen, and stored at -80°C for later use.

For ALFA-tagged GIRK, full-length mouse GIRK2 with a C-terminal ALFA peptide tag upstream of a GFP tag was expressed using HEK293S GnTI$^-$ cells (ATCC) at a density of ~3.5*10^6 cells/mL infected with 10% (v/v) P3 virus. 10 mM sodium butyrate was added 12 hours after infection and the temperature was reduced to 30°C for 48 hours.

Cells were harvested by centrifugation at 3,500 x g for 15 minutes and pellets were flash frozen and stored at -80°C until use. Purification was carried out at 4°C. ~30 mL of cells were resuspended in buffer containing 25 mL Tris pH 7.5, 150 mM KCl, 2 mM DTT, DNase and protease inhibitors (12.5 $\mu g/mL$ leupeptin, 12.5 $\mu g/mL$ pepstatin A, 625 $\mu g/mL$ AEBSF, 1 mM Benzamadine, 100 $\mu g/mL$ Trypisin inhibitor, 1x aprotinin, and 1 mM PMSF) and lysed by manual homogenization. Membranes were separated by centrifugation at 39,000 x g for 30 minutes, resuspended in buffer containing 20 mM Tris pH 7.5, 150 mM KCl, 2 mM DTT, and manually homogenized. Channels were extracted with 1.5% DDM/0.3% CHS for 1.5 hours and lysate was clarified by centrifugation 39,000 x g for 30 minutes. Lysate was bound to GFP nanobody-coupled Sepharose resin (prepared in house) for one hour, washed in batch one time with 10 column volumes of buffer containing 20 mM Tris pH 7.5, 150 mM KCl, 2 mM DTT, and 0.05%/0.01% DDM/CHS, then loaded into a column and washed with an additional 10 column volumes by gravity flow. Protein was eluted by cleavage with 3C PreScission protease (prepared in-house) for two hours, concentrated to ~10 mg/mL using a 15-mL Amicon concentrator with 100-kDa molecular weight cutoff, and further purified by size exclusion chromatography using a Superose 6 10/300 increase column in buffer containing 20 mM Tris pH 7.5, 150 mM KCl, 10 mM DTT, and 0.025%/0.005% DDM/CHS. Fractions with GIRK2 were pooled, concentrated to 2 mg/mL and used for reconstitution immediately.

Protein Reconstitution

GIRK and lipidated $G\beta\gamma$ were reconstituted for bilayer experiments using 3:1 ratio of 1-palmitoyl-2-oleoyl-sn-glycero-3-phosphoethanolamine (POPE): 1-palmitoyl-2-oleoyl-sn-glycero-3-phospho-(1'-rac-glycerol) (POPG) lipids. Lipids in chloroform were mixed and dried under a stream of argon, washed with pentane, dried under a stream of argon and incubated under vacuum overnight. Lipids were resuspended at 20 mg/mL in buffer containing 10 mM K_2HPO_4, 450 mM KCl, and 10 mM DTT and sonicated to clarity. 1% DM was added to the lipids and the mixture was sonicated extensively. GIRK was added to the mixture at a protein to lipid ratio of 1:10 (wt/wt), or $G\beta\gamma$ was added at 1:5 (wt/wt), the lipid concentration was diluted to 10 mg/mL and incubated at 4°C for one hour. Detergent was removed at 4°C using dialysis in a tubing with a 50 kDa cutoff for GIRK or a 10 kDa cutoff for $G\beta\gamma$ in buffer containing 10 mM K_2HPO_4 pH 7.4, 450 mM KCl, and 10 mM DTT, and biobeads. Dialysis buffer was changed every 12 hours for three changes with fresh DTT added at each change. After 3 changes, liposomes were incubated with ~50% volume of biobeads for five hours at 4°C, flash frozen, and stored at -80°C until use.

For structural studies using liposomes, $G\beta\gamma$ was reconstituted using a 2:1:1 mixture of 1,2-dioleoyl-sn-glycero-3-phosphoethanolamine (DOPE): 1-palmitoyl-2-oleoyl-glycero-3-phosphocholine (POPC): 1-palmitoyl-2-oleoyl-sn-glycero-3-phospho-L-serine (POPS). Lipids in chloroform were mixed and dried under a stream of argon, washed with pentane, dried under a stream of argon, and incubated under vacuum overnight. Lipids were resuspended at 25 mM in buffer containing 25 mM HEPES pH 7.4, 150 mM KCl and 5 mM DTT and sonicated to clarity. 40 mM sodium cholate was added and the mixture was sonicated briefly. $G\beta\gamma$ was added at a protein to lipid ratio of

1:15 (wt/wt) and the lipid concentration was reduced to 20 mM maintaining 40 mM sodium cholate and incubated at 4°C for one hour. Detergent was removed using four exchanges of 200 mg/mL biobeads washed with reconstitution buffer after two hours, 12 hours, two hours, and two hours at 4°C. Liposomes were used immediately following reconstitution to prepare grids. Reconstitution for partition experiments was carried out in the same way but 0.1 $mole\%$ 1,2-dioleoyl-sn-glycero-3-phosphoethanolamine-N-(lissamine rhodamine B sulfonyl) (18:1 Liss Rhod PE) was included, the final lipid concentration was diluted to 12.5 mM, and $G\beta\gamma$ was added at a protein to lipid ratio of 1:5 (wt/wt). Liposomes for partitioning studies were flash frozen and stored at -80°C until use.

For nanodiscs, 2DOPE:1POPC:1POPS lipids were used. Lipids in chloroform were mixed and dried under a stream of argon, washed with pentane, dried under a stream of argon, and incubated under vacuum overnight. Lipids were resuspended at 20 mM in buffer containing 25 mM HEPES pH 7.4, 150 mM KCl, 5 mM DTT, and 40 mM sodium cholate and sonicated to clarity. MSP2N2 scaffold (10), $G\beta\gamma$-YFP, and lipids, were mixed at a molar ratio of 1:0.75:220 in buffer containing 25 mM HEPES pH 7.4, 150 mM KCl, and 5 mM DTT and incubated for one hour at 4°C. Detergent was removed using two incubations with 200 mg/mL biobeads washed with reconstitution buffer changed after two hours and 12 hours. Harvested nanodiscs were diluted to 3 mL with buffer containing 5 mM HEPES pH 7.4, 150 mM KCl, and 5 mM DTT and bound in batch to GFP nanobody-coupled Sepharose resin (prepared in house) for one hour. Resin was loaded onto a column and washed with 10 column volumes of 25 mM HEPES pH 7.4, 150 mM KCl, and 5 mM DTT buffer by gravity flow and eluted by cleavage with 3C PreScission protease (prepared in-house) for two hours. Nanodiscs were concentrated to 1 mL using a 4-mL Amicon concentrator with 100-kDa molecular weight cutoff, and further purified by size exclusion chromatography using a Superose 6 10/300 increase column in buffer containing 25 mM HEPES pH 7.4, 150 mM KCl, and 5 mM DTT. Reconstitution of $G\beta\gamma$ was confirmed using SDS-PAGE, nanodiscs were concentrated to 5 mg/mL using a 0.5-mL Amicon concentrator with 100-kDa molecular weight cutoff and used immediately for grid preparation.

Bilayer experiments and analysis

A lipid mixture of 2DOPE:1POPC:1POPS with various concentrations of L-α-phosphatidylinositol-4,5-bisphosphate (Brain, Porcine) ($PIP2$) was used. Lipids in chloroform were dried under a stream of argon and resuspended at 20 mg/mL in decane. A vertical bilayer configuration was used for all experiments. Two chamber cups (3 mL each) were connected by a 100 μm thick piece of Fluorinated ethylene propylene copolymer with a ~250 μm hole, which was used to paint a bilayer with the lipid-decane mixture. Buffer containing 25 mM HEPES pH 7.4, 150 mM KCl, 30 mM NaCl, 2 mM $MgCl_2$, and 100 μM $CaCl_2$ was used in both chambers. The reference electrode and the ground electrode were connected to the trans and cis chambers respectively via agarose salt bridges. A magnetic stir bar was included in the cis chamber to facilitate mixing. Voltage across the lipid bilayer was controlled with an Axopatch 200Bamplifier in whole-cell mode. The analog current signal was lowpass filtered at 1 kHz (Bessel) and digitized at 10 kHz with a Digidata 1440A digitizer. Digitized data were recorded with

software pClamp (MolecularDevices). 30 nM Nanobody-tagged $G\beta\gamma$ was added at the beginning of each experiment. All reagents (GIRK-containing vesicles, $PLC\beta3$, etc) were added to the cis chamber under continuous mixing. Frozen GIRK-containing liposomes were thawed on ice and KCl solution was added to a final concentration of 1 M. Before fusing liposomes, they were sonicated briefly at room temperature.

For the $PIP2$ titration experiments, the starting concentration of $PIP2$ in the bilayer was varied from 0.1 $mol\%$ to 4 $mol\%$. For each experiment, GIRK-containing vesicles were fused and current at +80 mV was measured. While recording, 32 μM C8PIP2 was added to maximally activate the channels. The starting current was normalized to the maximal current and reported as I/Imax. Each starting $PIP2$ concentration was repeated at 3-6 times. For $PLC\beta3$ experiments, 1 $mol\%$ $PIP2$ was used. After fusing vesicles with GIRK, baseline current at +80 mV was measured for 3-5 minutes and then 29 nM (1x) or 58 nM (2x) $PLC\beta3$ was added to the cis chamber while recording. After the current decay, a voltage family was measured to ensure integrity of the bilayer and then 32 μM $C8PIP2$ was added to recover channel activity. Any experiment where the current did not recover was discarded. For experiments with soluble $G\beta\gamma$, 1 μM was added before the addition of $PLC\beta3$. We are confident that the starting PIP2 concentration in the membrane was not affected by vesicle fusion for two reasons. First, our titration experiments (Fig 2A) show consistent results for each starting PIP2 concentration and do not depend on the number of channels in the bilayer. Second, the kinetics of PIP2 hydrolysis are also not dependent on the number of channels that fused.

Analysis was carried out in Clampfit. To reduce the amplitude of high-frequency undesired signal owing largely to the stir bar in the recording chamber, current time series were low pass filtered at 20 Hz, then down-sampled by a factor of 10 (experiments with $G\beta\gamma$) or 100 (experiments without $G\beta\gamma$) and exported to qtiplot. We also analyzed our fastest decays without low pass filtering to ensure that the 20 Hz filter did not alter the determination of V_{max} and K_M to a significant degree (i.e., 20 Hz did not over filter the kinetic process under study).

In the data presented in Fig. 2A-D, the leak was subtracted and the decays were normalized to the starting $PIP2$ concentration and converted to $PIP2$ decays using Equation 1. For direct fitting of normalized current decays to determine K_M and V_{max}, current decays were normalized to the starting $PIP2$ concentration and directly fit to Equation 4 to determine K_M and V_{max}. Because $I_{GIRK(max)} \gg I_{leak}$, we fit the data to Equation 4, where C represents $\frac{I_{leak}}{I_{GIRK(max)}}$.

NMR experiments to measure lipid concentration

A small volume of liposomes from reconstitutions (10-20 μL) was dissolved in ~550 uL of a mixture of deuterated methanol and chloroform (5:1 or 4:1) containing 100 μM of the standard sodium trimethylsilyl propionate (TSP). Proton spectra were measured on a Bruker 600 MHz instrument equipped with an AVANCE NEO console and a 5 mm HCN cryoprobe. Spectra were collected in 5 mm tubes at 298 K using a 30° flip angle, 16 scans, and 2.8 second acquisition time and a recycle delay of 18 μs. Spectra were processed using TopSpin 4.1.1 for line broadening, phasing, and baseline correction. The

triplet lipid -CH_3 peak at 0.875 ppm was integrated relative to the TSP peak at 0 ppm and normalized to the difference in protons (nine for TSP and 6 for the lipid CH_3) (Figure S2A). The normalized peak area was used to determine the lipid concentration using the known 100 μM concentration of TSP.

PLCβ vesicle partition experiments

Reconstituted liposomes with or without $G\beta\gamma$ were subjected to 10 freeze-and-thaw cycles and extruded 21 times through a 200 nm membrane to produce LUVs. Fixed concentrations of lipids were mixed with LD655-labeled $PLC\beta3$ in buffer containing 25 mM HEPES pH 7.4, 150 mM KCl, and 5 mM DTT, incubated for one hour, and centrifuged for one hour at 100,000 x g at room temperature. The supernatant was removed and the membrane pellet was resuspended in an equal volume of buffer. The input, pellet, and supernatant samples were analyzed by SDS-PAGE and imaged using in-gel fluorescence to detect LD655-labeled $PLC\beta$ (Figure S2D-E). Gel bands were quantified using Bio-Rad imagelab software. Additionally, input, supernatant, and pellet samples were solubilized in 3-5% Anapoe-C12E10 to eliminate scattering artifacts and the fluorescence signal was measured for LD655 (ext-649, em-666) and Rhodamine (ext-560, em-583) using a Tecan plate reader. The Rhodamine signal was used to estimate the fraction of lipids that were pelleted and the measurements were corrected for this as well as the loss of material using the difference between the input and output (pellet and supernatant) LD655 signal. The reported lipid concentration is 50% of the total lipid concentration added in solution because $PLC\beta3$ only has access to the outer leaflet. Each lipid concentration was repeated with two different $PLC\beta3$ concentrations, 100 nM and 300 nM. Additional experiments with unlabeled 200 nM and 300 nM $PLC\beta3$ were carried out to confirm that the fluorescent label does not alter the partitioning behavior of $PLC\beta3$. These experiments were analyzed using SDS-PAGE and quantified using Bio-Rad stain free gels (Figure S2B-C). There was no difference between labeled and unlabeled $PLC\beta3$ but the fluorescence quantification was more robust so we used these values for our calculations.

In the absence of $G\beta\gamma$, values for K_x were determined for each experiment using Equation 5 and values for fraction of $PLC\beta3$ partitioned (F_p), were determined, plotted against lipid concentration, and fit to Equation 6 for a more robust determination of K_x (Figure 3D, S2F-G). In the presence of $G\beta\gamma$, values for K_x^{App} were determined for each experiment using Equation 5 and values for fraction of $PLC\beta3$ partitioned (F_p), were determined, plotted against lipid concentration, and fit to Equation 7 for K_{eq} (Figure 3D, S2A-B). Values from the gels and the solution fluorescence measurements were consistent and the values from the solution measurements are reported. Individual values for K_x and K_x^{App} do not vary with $PLC\beta3$ or lipid concentrations, as expected (1). Further, values of F_p, K_x, and K_x^{App} were consistent across multiple reconstitutions and preparations (Figure S2F-G).

Cryo-EM sample preparation and data collection

For $PLC\beta3$ in solution without liposomes, glycerol was omitted from the size exclusion buffer and protein was concentrated to 4.8 mg/mL using a 4-mL Amicon concentrator with 100-kDa molecular weight cutoff. Protein was supplemented with 3 mM of Fluorinated Fos-Choline-8 ~5 minutes before grid preparation. Quantifoil R1.2/1.3 400 mesh holey carbon Au grids were glow discharged for 20s, and 3.5 μL of sample was applied. After 20s incubation at 16°C and 100% humidity, grids were blotted for 2.5s with a blot force of 0, and plunge frozen in liquid ethane using a FEI Vitrobot Mark IV. For data acquisition, grids were loaded onto a 300-kV Titan Krios transmission electron microscope with a Gatan K3 Summit direct electron detector and a GIF quantum energy filter with a slit width of 20 eV and 3,527 movies were collected in superresolution mode with a pixel size of 0.54 Å and a defocus range of 1 to 2.5 μm using SerialEM (11). The movies were recorded with 40 frames, a 2 second total exposure time (0.05s/frame) and a dose rate of 25 e^-/pix/s which gave a cumulative dose of 42.87 e^-/Å^2 (1.07 e^-/Å^2/frame).

For $PLC\beta3$ and reconstituted liposomes without $G\beta\gamma$, $PLC\beta3$ and liposomes were mixed at final concentrations of 0.5 mg/mL and 17.5 mM respectively and incubated at room temperature for one hour. The sample was supplemented with 3 mM of Fluorinated Fos-Choline-8 ~5 minutes before grid preparation. Quantifoil R1.2/1.3 400 mesh holey carbon Au grids were glow discharged for 20s, and 3.5 μL of sample was applied, incubated for 5 minutes at 20°C and 100% humidity, and manually blotted from below. An additional 3.5 μL of sample was applied and after 30 seconds of incubation, grids were blotted for 3s with a blot force of 0, and plunge frozen in liquid ethane using a FEI Vitrobot Mark IV. For data acquisition, grids were loaded onto a 300-kV Titan Krios transmission electron microscope with a Gatan K3 Summit direct electron detector and a GIF quantum energy filter with a slit width of 20 eV and 5,448 movies were collected in superresolution mode with a pixel size of 0.435 Å and a defocus range of 1.5 to 2.5 μm using SerialEM (11). The movies were recorded with 50 frames, a 1.5 second total exposure time (0.05s/frame) and a dose rate of 25 e^-/pix/s which gave a cumulative dose of 50.7 e^-/Å^2 (1.01 e^-/Å^2/frame).

For $PLC\beta3$ and reconstituted liposomes with $G\beta\gamma$, $PLC\beta3$ and liposomes with 1:15 (wt/wt) $G\beta\gamma$ were mixed at final concentrations of 0.5 mg/mL and 17.5 mM respectively and incubated at room temperature for one hour. The sample was supplemented with 3 mM of Fluorinated Fos-Choline-8 ~5 minutes before grid preparation. Quantifoil R1.2/1.3 400 mesh holey carbon Au grids were glow discharged for 20s, and 3.5 μL of sample was applied, incubated for 5 minutes at 22°C and 100% humidity, and manually blotted from below. An additional 3.5 μL of sample was applied and after 30 seconds of incubation, grids were blotted for 3.5s with a blot force of 0 and plunge frozen in liquid ethane using a FEI Vitrobot Mark IV. For data acquisition, grids were loaded onto a 300-kV Titan Krios transmission electron microscope, located at the HHMI Janelia Research Campus, with a Gatan K3 Summit direct electron detector and a GIF quantum energy filter with a slit width of 20 eV and 27,454 movies were collected in superresolution mode with a pixel size of 0.4195 Å and a defocus range of 1.5 to 2.5 μm using SerialEM (11). The movies were recorded with 50 frames, a 4.194 second total exposure time (0.084s/frame) and a total dose of 60 e^-/Å^2 (1.2 e^-/Å^2/frame).

For $PLC\beta3$ and $G\beta\gamma$ reconstituted into nanodiscs, nanodiscs were concentrated to 4 mg/mL using a 0.5-mL Amicon concentrator with 100-kDa molecular weight cutoff, mixed with $PLC\beta3$ to final concentration of 3.5 mg/mL nanodiscs and 2.5 mg/mL

$PLC\beta$ and incubated at room temperature for one hour. The sample was supplemented with 3 mM of Fluorinated Fos-Choline-8 ~5 minutes before grid preparation. Quantifoil R1.2/1.3 400 mesh holey carbon Au grids were glow discharged for 20s, and 3.5 μL of sample was applied. After 20s incubation at 16°C and 100% humidity, grids were blotted for 2.5s with a blot force of -3, and plunge frozen in liquid ethane using a FEI Vitrobot Mark IV. For data acquisition, grids were loaded onto a 300-kV Titan Krios transmission electron microscope with a Gatan K3 Summit direct electron detector and a GIF quantum energy filter with a slit width of 20 eV and 25,063 movies were collected in counting mode with a pixel size of 0.844 Å and a defocus range of 1 to 2.5 μm using Leginon (12). The movies were recorded with 40 frames, a 2 second total exposure time (0.05s/frame) and total dose of 69.14 $e^-/Å^2$ (1.72 $e^-/Å^2$/frame).

Cryo-EM data processing

For $PLC\beta$3 in solution without liposomes, motion correction was performed using MotionCorr 2 with 2x binning via RELION (in RELION 3.1) and contrast transfer function (CTF) estimation was carried out using CTFfind4 (13-15). 1,489,175 particles were picked using Laplacian-of-Gaussian (LoG) picking in RELION 3.1, extracted without binning with a box size of 211.7 Å, and imported into cyroSPARC (15, 16). 505,209 particles were selected after several rounds of 2D classification and used to generate an initial model (*ab initio* reconstruction), which resembled the crystal structure of PLCβ3 with density for both the catalytic core and the distal CTD. 195,277 particles were selected from heterogenous refinement using the PLCβ3 initial model and two junk models and imported into RELION 3.1using the csparc2star.py script (17) for an additional round of 3D classification with global alignment, which yielded a subset of 136,521 particles. These particles were subjected to 2D classification in RELION ignoring the CTF until the first peak to generate templates for auto-picking. Micrographs with an estimated resolution worse than 7 Å were discarded and the remaining 2,477 were used for template-based picking resulting in 1,021,849 particles, which were extracted with a box size of 211.7 Å. These particles were sorted with several rounds of 2D classification in cryoSPARC2 resulting in 771,044 particles, which were subjected to *ab initio* reconstruction with 5 classes: two junk classes, two classes resembling the PLCβ3 crystal structure with density for the catalytic core and the distal CTD, and one class resembling the PLCβ3 catalytic core. These particles were then subjected to heterogenous refinement with five models including one junk model, three models containing density for both the catalytic core and distal CTD and one containing only density for the catalytic core. 263,592 particles were sorted into the class with just the catalytic core, imported into RELION 3.1 using the csparc2star.py script (17), and refined to 4.3 Å. The remaining particles yielded low resolution reconstructions of PLCβ3 with varying amounts of density for the catalytic core and distal CTD. Extensive 3D classification with and without alignment including focused classification did not improve reconstructions with both domains, even with a larger box size of 276.5 Å. The 263,592 particles in the catalytic core reconstruction were subjected to two rounds of Bayesian particle polishing (13) and the resolution improved to 4 Å. To ensure that box size was not too small to accommodate the distal CTD, these particles were re-extracted with a box size of 276.5 Å and subjected to an additional three rounds of Bayesian

particle polishing (18) and one round of CTF refinement (13) resulting in a 3.8 Å reconstruction. Attempts to recover density for the distal CTD from the reconstruction were unsuccessful. To improve the resolution, one round of masked 3D classification without alignment was carried out, resulting in the selection of 67,716 particles which refined to 3.6 Å. Resolution was evaluated using the 0.143 criterion of the Fourier shell correlation trough the PostProcess job in RELION (19) (Figure S3). This map was used for model building.

For $PLC\beta3$ alone on vesicles, motion correction was performed using MotionCorr 2 with 2x binning via RELION (in RELION 3.1) and contrast transfer function (CTF) estimation was carried out using CTFfind4 (13-15). ~1,100 particles were manually picked from 23 micrographs in crYOLO (3) and used to train a new model for picking (Figure S4A-C). 244,212 particles were picked, manually inspected to ensure integrity of picks with the new model, extracted with 2x binning and a box size of 222.7 Å in RELION 3.1, and imported into cryoSPARC (13, 16). 190,186 particles were selected after two rounds of sorting with 2D classification and subjected to *ab initio* reconstruction with three classes, one of which resembled $PLC\beta3$ with density for both the distal CTD and the catalytic core. Particles were sorted with a resolution cutoff of 5 Å (206,044 particles remaining) and refined to this model to attempt to align the membranes yielding the reconstruction shown in Figure S4C. The particles were subjected to extensive 2D and 3D classification with and without alignment in RELION 3.1 and cryoSPARC, with and without signal subtraction of the membrane, and focused classification on the individual domains but no higher resolution reconstructions were obtained (Figure S4).

For the $PLC\beta3/G\beta\gamma$ complex on liposomes, motion correction was performed with 2x binning using the RELION implementation (in RELION 3.1) and CTF estimation was carried out using CTFfind4 (13-15). 2,411,476 particles were picked using the model trained for $PLC\beta3$ on vesicles in crYOLO (3) and sorted with an estimated resolution cutoff of 5.5 Å, resulting in 1,997,424 particles. A subset of 416,198 particles from 3,516 micrographs were extracted with 2x binning and a box size of 214.8 Å and imported into cryoSPARC3 for initial analysis (13, 16). 337,132 particles were selected from sorting with 2D classification and subjected to *ab initio* reconstruction with four classes. One class with 66,007 particles yielded a low resolution reconstruction showing density for the membrane and external protein. All 416,198 particles were refined to this model to attempt to align the membranes, imported into RELION 3.1 using the csparc2star.py script (17) and subjected to 2D classification without alignment ignoring the CTF until the first peak. 32,673 particles from 2D averages showing two features on the membrane surface were selected, imported into cryoSPARC3 and subjected to *ab initio* reconstruction with two classes (Figure S5). One class yielded a model with membrane density and two external features. All 1,997,424 particles were extracted with 2x binning and a box size of 255 Å and refined to this model in RELION to attempt to align the membranes. The membrane density was subtracted and the particles were subjected to several rounds of 3D classification. ~400,000 particles populated classes that resembled the $PLC\beta3$ catalytic core bound to two $G\beta\gamma$s and 121,394 particles from one class produced a reconstruction with clear secondary structure features. These particles were reverted to the original coordinates to undo the signal subtraction, re-extracted without binning and a box size of 289 Å, and imported into cryoSPARC3 for further analysis.

Non-uniform refinement yielded a 4.4 Å reconstruction and included strong density for the membrane. Local refinement with a mask on the protein complex yielded a 3.9 Å reconstruction of the protein complex. These particles were subjected to Bayesian particle polishing and CTF refinement in RELION 3.1 (13, 18) and refined again in cryoSPARC3 resulting in a 3.8 Å reconstruction from non-uniform refinement and a 3.5 Å reconstruction from local refinement with a mask on the protein complex. The resolution was evaluated using the 0.143 criterion of the Fourier shell correlation in cryoSPARC3 (19). The map from local refinement was used for model building. These particles were subjected to additional 2D and 3D classification without alignment in RELION to investigate the position of the complex on the membrane. Resulting 3D classes were refined in cryoSPARC3 using non-uniform refinement and used for subsequent analysis (Figure 7, S5).

For the $PLC\beta \cdot G\beta\gamma$ complex on nanodiscs, motion correction was performed using the RELION implementation (in RELION 3.1) and CTF estimation was carried out using CTFfind4 (13-15). 3,993,932 particles were picked using crYOLO with the general model (3), imported into RELION 3.1 and extracted 2x binned with a box size of 324 Å. Particles were sorted with a 5 Å estimated resolution cutoff in RELION resulting in 2,845,571 particles, which were imported into cryoSPARC3 and further sorted with 2D classification (13, 16). An initial model was determined using 1,166,729 particles following 2D classification, which resembled the $PLC\beta3$ catalytic core bound to two $G\beta\gamma$s. These particles were refined to the initial model resulting in a low resolution reconstruction. Focused 3D classification on the $PLC\beta$ catalytic core and $G\beta\gamma2$ was carried out in RELION 3.1, which yielded a reconstruction with 947,408 particles with secondary structure features. The model was improved by iterative 3D classification in RELION 3.1 and homogenous refinement in cryoSPARC3, which yielded a ~3.5 Å reconstruction from 55,623 particles. The map showed some artifacts from overfitting and over-sampled orientations. To improve the map quality and obtain more particles, sorting was repeated. The initial 2,845,571 particles were sorted to 2,428,922 particles with one round of 2D classification to remove obvious junk and subjected to heterogenous refinement in cryoSPARC3 with the map of the PLCβ3/Gβγ complex and two junk maps. 828,450 particles were sorted into the $PLC\beta3/G\beta\gamma$ complex class and refined to 4.2 Å. Iterative 3D classification with and without alignment in RELION 3.1 and cryoSPARC4 yielded a subset of 168,057 particles which refined to 3.8 Å using non-uniform refinement with improved map quality. These particles were re-extracted without binning and underwent two rounds of Bayesian particle polishing in RELION (18) resulting in a 3.3 Å reconstruction from cryoSPARC3 non-uniform refinement. A final round of 3D classification without alignment in RELION resulted in a subset of 53,984 particles, which yielded a 3.3 Å reconstruction via local refinement in cryoSPARC3 with a mask on the protein complex. The resolution was evaluated using the 0.143 criterion of the Fourier shell correlation in cryoSPARC (19). This map was free of artifacts and was used for model building.

Model building and Validation

For $PLC\beta3$ in solution without liposomes, the crystal structure of $PLC\beta3$ catalytic core from the PDB 4GNK (4) was used as a starting model. It was fit into the density and

refined with PHENIX real-space refine (20) and manually inspected and adjusted where necessary. Regions with poor or weak density were removed. The proximal CTD (from 867 to 881) was excised from the crystal structure and fit into its density. The final model contains residues 12-92, 97-470, 574-850, and 867-881 and the side chains of E14, R169, K184, E191, K196, R199, E201, E232, K238, D777 were removed due to lack of density.

For the $PLC\beta3/G\beta\gamma$ complex on liposomes, the model for $PLC\beta3$ in solution was merged with two copies of $G\beta\gamma$ from PDB 4KFM (21) to use as the starting model. It was fit into the density and refined with PHENIX real-space refine (20) and manually inspected and adjusted where necessary. Regions with poor or weak density were removed. The final model consists of $PLC\beta3$: 13-92, 97-470, 576-849, 867-881 with the side chains of E14, E88, K184, E187, E191, R199, E201, R288, E303, R369, E373, E386, K603, R609, K631, K641, D777, K761, R872, and R874 removed due to lack of density, $G\beta1$: 4-126, and 133-340 with the side chains of R137, R214, E215, and R256 removed due to lack density, $G\gamma1$: 8-62, $G\beta2$: 2-126, and 133-340, with side chains of D292 and R52 removed due to lack of density, $G\gamma2$: 8-51.

For the $PLC\beta3/G\beta\gamma$ complex on nanodiscs, the model for the complex on liposomes was used as the starting mode. It was fit into the density and refined with PHENIX real-space refine (20) and manually inspected and adjusted where necessary. Regions with poor or weak density were removed. The final model consists of $PLC\beta3$: 13-92, 97-470, 576-849, 867-881 with the sidechains of E14, D71, K82, E88, K184, E303, E373, D777 removed due to lack of density, $G\beta1$: 4-126, and 133-340 with sidechains removed from R214, E215, M216, Q259 due to lack of density, $G\gamma1$: 8-62, $G\beta2$: 2-126, and 133-140 with sidechains removed from : D153, Q175, R191, R256 due to poor density, and $G\gamma2$: 8-51. Model quality was assessed with validation in Phenix using MolProbity score (22) and geometry evaluation. Figures were made using ChimeraX (23, 24).

For all proteins, sidechains were not removed in areas of week density due to prior knowledge of structure of each component.

SI Appendix 2: Derivation of Equation 7

In[∘]:=

```
(* DERIVATIONS IN MATHEMATICA CODE. THIS IS EXECUTABLE *)

(* ORIGIN OF EQ (7) *)

(* units of W,
L and PLCtot are in moles (or molar if we divide by solution volume),
all in else mole fraction. mole fraction is
 approximated as moles solute/moles sovent. So, for example,
PLCm*L = (moles of PLCm/ moles of lipid) * moles of lipid =
  moles PLCm. PLCm = PLC in membranes (lipids),
PLCGm = PLC bound to Gbg (which is in lipids) and PLCw = PLC in water *)
(* starting equations:
   Keq = Ggb*PLCm/PLCGm,
Kx = PLCm/PLCw,
Fp = moles of PLC in lipid / moles of PLC in lipid + moles of PLC in water =
  (PLCm + PLCGm)*L/((PLCm + PLCGm)*L + (PLCw)*W)=
   (1 + PLCGm/PLCm)/((1 + PLCGm/PLCm) + (PLCw/PLCm)*W/L)=
    Kx(1 + Gbg/Keq)/(Kx(1 + Gbg/Keq) + (W/L)),
Gtot = Gbg + PLCGm,
PLCtot = PLCmmoles + PLCGmmoles + PLCwmoles = (PLCm + PLCGm)L + (PLCw)W
    Note we can divide through this last equation by the experimental vessel
    volume and thus use molar units for PLCtot, W and L in our equations.

  Our aim is to express Fp(PLCtot,Gtot,Kx,Keq,L,W). We want to do
 this because we know PLCtot, Gtot, L and W from experimental setup
 (note we are expressing PLCtot, L and W in molar units and Gtot in mole fraction)
 and we determined Kx in experiment without Gbg. Our only unknown then is Keq,
which will be our single free parameter when we
 fit Fp to the partition data with Gbg.  *)

(* 1: solve PLCGm from Keq = Ggb*PLCm/PLCGm (all mole fraction units) *)
Solve[Keq == Ggb * PLCm / PLCGm, PLCGm]
```

$$\left\{\left\{PLCGm \to \frac{Ggb\ PLCm}{Keq}\right\}\right\}$$

In[∘]:=

```
(*2: sub PLCGm from 1 into PLCtot definition
  (L and W are molar, times mole fraction gives molar for PLCtot) *)
```

$$(PLCm + PLCGm)\ L + (PLCw)\ W\ /.\ PLCGm \to \frac{Gbg\ PLCm}{Keq}$$

In[•]:=
```
(* 3: sub PLCGm from 1 into Gtot definition (all mole fraction units) *)
Gbg + PLCGm /. PLCGm → (Ggb PLCm)/Keq
```

In[•]:=
```
(* 4: from 3, solve for Gbg  (all mole fraction units) *)
Solve[Gtot == Gbg + (Gbg PLCm)/Keq, Gbg]
```

In[•]:=
```
(* 5: sub 4 into 2 to eliminate Gbg from def of PLCtot
   (L and W molar, all else mole fraction to give PLCtot in molar) *)
L (PLCm + (Gbg PLCm)/Keq) + PLCw W /. Gbg → (Gtot Keq)/(Keq + PLCm)
```

In[•]:=
```
(* 6: sub PLCw->PLCm/Kx into 5 to get PLCtot ->
   f(knowns,PLCm,keq) (L,W molar, else mole fraction) *)
L (PLCm + (Gtot PLCm)/(Keq + PLCm)) + PLCw W /. PLCw -> PLCm / Kx
```

In[•]:=
```
(* 7: from 6 solve for PLCCm ->
  f(knowns, keq) (This gives PLCtot in molar units as a function
     of molar and dimensionless (mole fraction) variables. Note
     that each term is dimensionless after dividing through) *)
Solve[PLCtot == L (PLCm + (Gtot PLCm)/(Keq + PLCm)) + (PLCm W)/Kx, PLCm]
```

In[•]:=
```
(* 8: sub the appropriate root of 7 into 4 to get Gbg ->
  f(knowns, Keq) (Gbg comes out dimensionless (mole fraction)) *)
(Gtot Keq)/(Keq + PLCm) /. PLCm -> 1/(2 (Kx L + W)) (-Gtot Kx L - Keq Kx L + Kx PLCtot - Keq W +
    Sqrt[-4 Keq Kx PLCtot (-Kx L - W) + (-Gtot Kx L - Keq Kx L + Kx PLCtot - Keq W)^2])
```

In[•]:=
```
(* 9: sub 8 into Fp equation to get Fp -> f(knowns, Keq) *)
fractioninmembrane = Simplify[Kx (1 + Gbg / Keq) / (Kx (1 + Gbg / Keq) + (W / L)) /. Gbg ->
   (Gtot Keq)/(Keq + (-Gtot Kx L-Keq Kx L+Kx PLCtot-Keq W+ Sqrt[-4 Keq Kx PLCtot (-Kx L-W) + (-Gtot Kx L-Keq Kx L+Kx PLCtot-Keq W)^2])/(2 (Kx L+W)))]
```

In[•]:=
```
(* EQ (7) Q.E.D. *)
```

In[•]:=
```
(* EXAMINE THE INFLUENCE OF Keq FOR A FIXED
 Gbg CONCENTRATION ON THE MEMBRANE FRACTION OF PLCb *)
```

In[•]:=
```
Ftoplot = fractioninmembrane /. PLCtot -> (29 * 10 ^ -9) /. Kx -> 2.9 * 10 ^ 4 /.
     Gtot -> 0.0033 /. W -> 55;

(* This plot shows how the value of Keq influences Fp[L]. Keq ->
 100 corresponds to infinity. *)
```

In[•]:=
```
Plot[{Ftoplot /. Keq -> 9 * 10 ^ -5, Ftoplot /. Keq -> 10 ^ -3,
   Ftoplot /. Keq -> 10 ^ -2, Ftoplot /. Keq → 1000}, {L, 0, 0.003},
 PlotRange -> {{0, 0.003}, {0, 1}}, AxesLabel → {"[L]", Fp},
 PlotLabels → Placed[{"9*10^-5", "10^-3", "10^-2", "1000"} ]]
```

Out[•]=

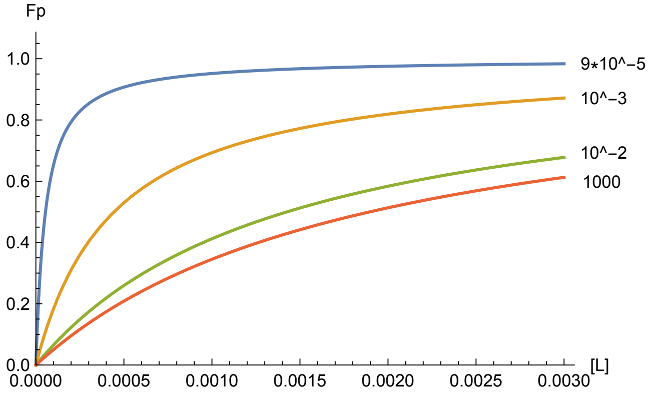

In[•]:=
```
(* ORIGIN OF EQ (8) *)

(* The bilayer case with large aqueous reservoir such that PLCm =
 Kx PLCw = constant *)
(* for Gtot = 0, PLCm =
 Kx PLCw = constant (i.e., we set PLCw experimentally). for Gtot > 0,
PLCmtot (not to be confused with PLCtot) =
 PLCm + PLCGm. From Gtot = Gbg + PLCGm and Keq = Ggb*PLCm/PLCGm =
    (Gtot - PLCGm)*PLCm/PLCGm have PLCGm = Gtot*PLCm/(Keq + PLCm). Therefore,
PLCmtot = PLCm + Gtot*PLCm/(Keq + PLCm) =
  PLCm*(1 + Gtot/(Keq + PLCm)) = Kx*PLCw*(1 + Gtot/(Keq + Kx*PLCw))
     Vmax = kcat*PLCmtot = Kx*PLCw*(1 + Gtot/(Keq + Kx*PLCw))*kcat *)
```

In[•]:=
```
(* EQ (8) Q.E.D. *)
```

SI References

1. S. H. White, W. C. Wimley, A. S. Ladokhin, K. Hristova, "[4] Protein folding in membranes: Determining energetics of peptide-bilayer interactions" in Methods in Enzymology. (Academic Press, 1998), vol. 295, pp. 62-87.
2. Y. Z. Tan *et al.*, Addressing preferred specimen orientation in single-particle cryo-EM through tilting. *Nature Methods* **14**, 793-796 (2017).
3. T. Wagner *et al.*, SPHIRE-crYOLO is a fast and accurate fully automated particle picker for cryo-EM. *Communications Biology* **2**, 1-13 (2019).
4. A. M. Lyon, S. Dutta, C. A. Boguth, G. Skiniotis, J. J. G. Tesmer (2013) Full-length Gαq-phospholipase C-β3 structure reveals interfaces of the C-terminal coiled-coil domain. in *Nature Structural and Molecular Biology* (Nature Publishing Group), pp 355-362.
5. M. R. Jezyk *et al.*, Crystal structure of Rac1 bound to its effector phospholipase C-β2. *Nature Structural & Molecular Biology* **13**, 1135-1140 (2006).
6. C. E. Ford *et al.*, Molecular basis for interactions of G protein βΓ subunits with effectors. *Science* **280**, 1271-1274 (1998).
7. A. Kirchhofer *et al.*, Modulation of protein properties in living cells using nanobodies. *Nature Structural & Molecular Biology* **17**, 133-138 (2010).
8. T. H. Charpentier *et al.* (2014) Membrane-induced allosteric control of phospholipase C-β isozymes. in *Journal of Biological Chemistry*, pp 29545-29557.
9. Q. Zheng *et al.*, Electronic tuning of self-healing fluorophores for live-cell and single-molecule imaging. *Chem. Sci.* **8**, 755-762 (2016).
10. Y. V. Grinkova, I. G. Denisov, S. G. Sligar, Engineering extended membrane scaffold proteins for self-assembly of soluble nanoscale lipid bilayers. *Protein Engineering, Design and Selection* **23**, 843-848 (2010).
11. D. N. Mastronarde, Automated electron microscope tomography using robust prediction of specimen movements. *Journal of Structural Biology* **152**, 36-51 (2005).
12. C. Suloway *et al.*, Automated molecular microscopy: The new Leginon system. *Journal of Structural Biology* **151**, 41-60 (2005).
13. J. Zivanov *et al.*, RELION-3 : new tools for automated high-resolution cryo-EM structure determination. *bioRxiv* 10.1101/421123, 1-38 (2018).
14. A. Rohou, N. Grigorieff, CTFFIND4: Fast and accurate defocus estimation from electron micrographs. *Journal of Structural Biology* **192**, 216-221 (2015).
15. S. Q. Zheng *et al.*, MotionCor2: Anisotropic correction of beam-induced motion for improved cryo-electron microscopy. *Nature Methods* **14**, 331-332 (2017).
16. A. Punjani, J. L. Rubinstein, D. J. Fleet, M. A. Brubaker, CryoSPARC: Algorithms for rapid unsupervised cryo-EM structure determination. *Nature Methods* **14**, 290-296 (2017).
17. D. Asarnow, E. Palovcak, Y. Cheng (2019) asarnow/pyem: UCSF pyem v0.5. (Zenodo).
18. J. Zivanov, T. Nakane, S. H. W. Scheres, A Bayesian approach to beam-induced motion correction in cryo-EM single-particle analysis. *IUCrJ* **6**, 5-17 (2019).
19. S. H. W. Scheres, S. Chen, Prevention of overfitting in cryo-EM structure determination. *Nature Methods* **9**, 853-854 (2012).

20. P. V. Afonine, J. J. Headd, T. C. Terwilliger, P. D. Adams, PHENIX News. *Computational Crystallography Newsletter* **4**, 43-44 (2013).
21. M. R. Whorton, R. MacKinnon, Crystal structure of the mammalian GIRK2 K + channel and gating regulation by G proteins, PIP 2, and sodium. *Cell* **147**, 199-208 (2011).
22. V. B. Chen *et al.*, MolProbity: All-atom structure validation for macromolecular crystallography. *Acta Crystallographica Section D: Biological Crystallography* **66**, 12-21 (2010).
23. E. F. Pettersen *et al.*, UCSF Chimera - A visualization system for exploratory research and analysis. *Journal of Computational Chemistry* **25**, 1605-1612 (2004).
24. E. F. Pettersen *et al.*, UCSF ChimeraX: Structure visualization for researchers, educators, and developers. *Protein Science* **30**, 70-82 (2021).
